# The physical chemistry of interphase loop extrusion

**DOI:** 10.1016/j.xgen.2025.101098

**Published:** 2025-12-10

**Authors:** Maxime M.C. Tortora, Geoffrey Fudenberg

**Affiliations:** 1 Department of Quantitative and Computational Biology, University of Southern California, Los Angeles, CA 90007, USA

**Keywords:** DNA loop extrusion, cohesin, genome folding, chromatin, Hi-C, biophysics, chemical-reaction network, biochemistry, molecular motor, polymer model

## Abstract

Cohesin drives genome organization via loop extrusion, orchestrated by the dynamic exchange of multiple essential accessory proteins. Although these regulators bind the core cohesin complex only transiently, their disruption can dramatically alter loop-extrusion dynamics and chromosome morphology. Still, a quantitative theory of cohesin regulation and its interplay with genome folding is still elusive. Here, we derive a chemical-reaction network model of loop-extrusion regulation from first principles that is fully specified by available *in vivo* measurements. This “bursty extrusion model” untangles the distinct roles of regulators, whose exchange coincides with intermittent periods of motor activity. By incorporating bursty extrusion in polymer simulations, we reveal how variations in regulatory protein abundance can alter chromatin architecture across length and timescales. Our results are corroborated by *in vivo* and *in vitro* observations, bridging the gap between cohesin-regulator dynamics at the molecular scale and their genome-wide consequences on chromosome organization.

## Highlights

• Chemical-reaction network model explicitly describes cohesin-regulator functions
• Cohesin undergoes bursty loop extrusion with alternating active and inactive states
• Rapid regulator exchange controls extrusion dynamics over much longer timescales
• Bursty extrusion model quantitatively relates cellular stoichiometry and genome folding

## Graphical abstract

**Figure:**
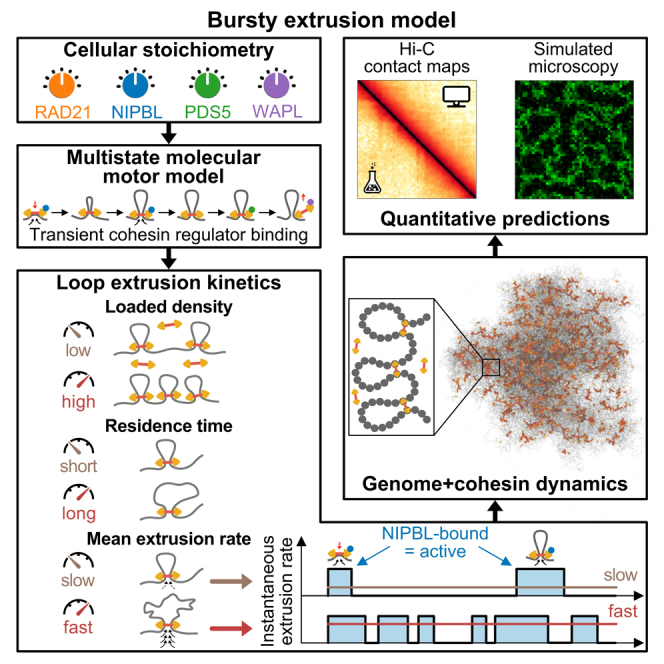

## Teaser

Tortora and Fudenberg derive a chemical-reaction network model explaining how cohesin regulators control loop extrusion from first principles. Experimental data uniquely support a “bursty extrusion” mechanism, where cohesins alternate between active and inactive phases during cycles of regulator exchange. Predictions of the bursty extrusion model are validated by microscopy and Hi-C, bridging molecular-scale cohesin dynamics with genome-wide structure.

## Introduction

Genomes are continuously and actively organized by loop extrusion.^1^ During this stochastic process, molecular motors load onto chromosomes and translocate to generate enlarging loops, until they eventually dissociate.^2^ Strong support for loop extrusion *in vivo* stems from the comparison of polymer model predictions with genomics data obtained from high-throughput chromosome conformation capture (Hi-C) experiments,^3^ as well as overall chromosome morphology in mitosis.^4^^,^^5^ More recently, *in vitro* single-molecule tracking assays provided direct evidence that the structural maintenance of chromosome (SMC) complexes cohesin and condensin can processively generate loops on tethered DNA molecules.^6^^,^^7^^,^^8^ Active modulation of loop-extrusion dynamics is now believed to regulate a growing number of cellular decision mechanisms. These range from controlling promoter choice at the protocadherin locus to modulate neural wiring,^9^ to V(D)J recombination to enable immune repertoire diversity.^10^

In mammalian interphase cells, cohesin acts as the main loop extruder.^11^ Increasing evidence argues that cohesin cannot be thought of as a monolithic complex and that transient associations with regulatory proteins modulate the dynamics of loop extrusion.^11^^,^^12^ The core cohesin complex consists of SMC1 and SMC3, one of SA1 or SA2, and the kleisin subunit RAD21. Among these core components, RAD21 acts as the “nexus”^13^ or “docking point”^14^ for the recruitment of cohesin-regulatory factors with multiple interfaces competent for cohesin-regulator binding. While many of these regulators were originally identified for their functions in sister chromatid cohesion,^15^^,^^16^ their roles for extrusion are now increasingly appreciated.^11^

Individual disruptions to the cohesin regulators NIPBL, PDS5, and WAPL can induce dramatic changes in cohesin properties and genome organization. The depletion of WAPL (i.e., ΔWAPL) leads to a considerable increase in cohesin residence times and loaded fraction on chromatin and results in the lengthwise compaction of entire chromosomes into “vermicelli,” which are highly enriched for cohesin along their axes.^17^ Vermicelli chromatids have a prophase-like appearance yet emerge from the action of interphase cohesin complexes. Similar phenotypes have been reported upon depletion of PDS5^18^^,^^19^ and overexpression of RAD21^20^ but are inhibited by the removal of NIPBL.^21^

At the molecular level, dissecting the respective functions of cohesin regulators poses a substantial challenge, due to the multiple roles reported for individual regulators. NIPBL has been suggested to act as a cohesin loader,^22^ but it is also required for ATP hydrolysis and translocation *in vitro*.^6^^,^^7^ PDS5 may facilitate cohesin unloading in conjunction with WAPL,^23^^,^^24^ but it also competes with NIPBL by binding with mutual exclusivity to the cohesin complex.^25^^,^^26^ While fine-grained quantitative models of SMC stepping have been proposed,^27^ quantitative descriptions of how cohesin regulators modulate loop extrusion are still elusive. For instance, although PDS5 has been recently identified as a “brake” for loop extrusion,^19^ a mathematical description of how the cohesin extrusion rate depends on the abundance of PDS5, or any other regulator, is currently lacking.

Despite their striking effects on loop-extrusion dynamics, biophysical measurements indicate that the residence time of NIPBL, WAPL, and PDS5 on chromatin (∼1 min^22^^,^^28^) is considerably less than that of cohesin (∼20 min for RAD21^17^^,^^29^^,^^30^). This dynamic turnover of regulators on the core cohesin complex implies that quantitative models of loop extrusion would benefit from depicting cohesins as multi-state motors with heterogeneous properties arising from the binding of distinct regulators.^31^ However, current extrusion models (1) lack a molecular basis for the roles of different cohesin regulators and (2) assume that all cohesins are single-state motors with identical extrusion behavior.^32^ Because of this, loop-extrusion parameters for existing models are obtained in part by fitting to match Hi-C data, rather than coming from first principles or biophysical measurements. Ultimately, new models are needed to incorporate insights from *in vitro* motor assays, account for roles of different cohesin regulators, and understand their downstream impacts on chromosome organization and genomic functions.

Chemical-reaction networks based on mass-action kinetics have proven highly successful for the modeling of a variety of other cellular regulatory processes.^33^ Still, they have not yet been leveraged to understand either loop extrusion or genome organization. Here, we derive a reaction network model governed by the stochastic exchange of cohesin regulators using available biophysical data from unperturbed cells. Our model describes cohesin as a multi-state molecular motor with “bursty” loop translocation kinetics derived from transient binding of regulators. In contrast with previous approaches, explicitly considering cohesin states bound by each regulator enables our model to predict both the changes in extrusion dynamics observed after depletions of cohesin regulators—as well as their consequences for 3D genome folding—directly from their abundances, without requiring any Hi-C data as input. Our model yields molecular insights into the differential roles of NIPBL, PDS5, and WAPL and provides a general framework for encoding biophysical data on cohesin and its regulators into computational models of loop extrusion. More broadly, our approach illustrates how quantitative measurements of chromatin association kinetics *in vivo* may be successfully used for the selection and parametrization of protein reaction networks to inform the design of biochemically realistic models of chromosome organization.

## Results

### Building a minimal biochemical-reaction network for interphase extrusion

We set out to develop a minimal model of interphase cohesin extrusion that nevertheless accounts for the individual roles of the key cohesin regulators NIPBL, PDS5, and WAPL, as well as the respective consequences of their disruption. This requires mathematically describing both the cohesin loading/unloading process along with the independent association and dissociation of cohesin with each of these three regulators. Thus, a multi-state model with *a minima* five distinct cohesin states is required to account for their separate influences on extrusion kinetics. We built our model using experimental data from HeLa cells, as, to our knowledge, this is the only cell line—in any organism—where comprehensive biophysical measurements of absolute abundance, bound fraction, and residence time have been made for each of these cohesin cofactors (STAR Methods). Leveraging the framework of mass-action kinetics, we made three main simplifying assumptions to describe transitions between cohesin states and derive a cohesin biochemical-reaction network: (1) RAD21 loading and unloading dynamics can be taken as a proxy for those of the core cohesin complex, (2) regulatory proteins require the core complex to be chromatin associated, and (3) regulators bind mutually exclusively to the core complex. Note that, while some of these approximations are likely too strong—e.g., they do not explicitly consider the possibility of a PDS5-WAPL complex on cohesin^34^^,^^35^—they yield a tractable description of the cohesin-regulatory network that we demonstrate is sufficient to recapitulate a broad range of experimental observations.

In our formalism, each network is characterized by a set of coupled ordinary differential equations and chemical-reaction rates quantifying the transitions between each state of the cohesin complex. Thus, the biochemical kinetics of a given cohesin network are fully determined by the values of the corresponding set of transition rates. Although not directly accessible to current experiments, we developed a general approach to exactly determine these rates from the experimentally measured chromatin-bound fraction and residence time of cohesin and its accessories based on analytical inversion of the coupled differential equations (see Rate mapping procedure in Methods S1). To that end, we curated experimental measurements of the *in vivo* abundance and kinetics of RAD21, NIPBL, WAPL, and PDS5 in unperturbed HeLa cells (amalgamating paralogs PDS5A and PDS5B; Table 1; STAR Methods). The bound fraction and residence times for each of the four proteins from fluorescence recovery after photobleaching (FRAP) enabled us to define and uniquely determine a rich zoo of minimal reaction networks with up to eight chemical transition rates (Methods S1, Rate mapping procedure). To identify the subset of these networks that are biologically relevant, we developed a two-stage pruning procedure. We first pruned networks with unphysical chemical kinetics (i.e., negative transition rates between states) to weed out reaction cycles that are incompatible with the experimental binding kinetics of each regulator. We further required that networks reproduce three qualitative observations for the bound fraction of RAD21 based on immunoprecipitation assays; specifically, (1) a decrease after ΔNIPBL, (2) an increase after ΔPDS5, and (3) an increase after ΔWAPL.^17^^,^^18^^,^^21^

**Table 1:** Absolute nuclear copy numbers, chromatin-bound fractions, and residence times of cohesin-associated proteins used to constrain the model in WT HeLa cells; see STAR Methods

Protein	Copy number	Chromatin-bound %	Chromatin residence time
RAD21	264,000^36^^,^^37^	65%^36^	822 s^36^
NIPBL	111,000^36^^,^^37^	40%^28^	72 s^28^
WAPL	65,000^36^^,^^37^	35%^22^	45 s^22^
PDS5A/B	164,000^38^	45%^22^	70 s^22^

The simplest class of cohesin reaction networks are completely reversible and acyclic and are characterized by linear, branched, and star topologies (Figure S1). However, none of these networks provided viable descriptions of cohesin biochemistry, as none of them survived pruning by the requirement for an increase in the bound fraction of RAD21 upon WAPL depletion (constraint (3)). We thus provide mathematical evidence that the chromatin entry and exit of cohesin via distinct molecular pathways applies not only for sister chromatid cohesion in S phase^15^^,^^16^ but also holds for loop extrusion in interphase.

**Figure 1:**
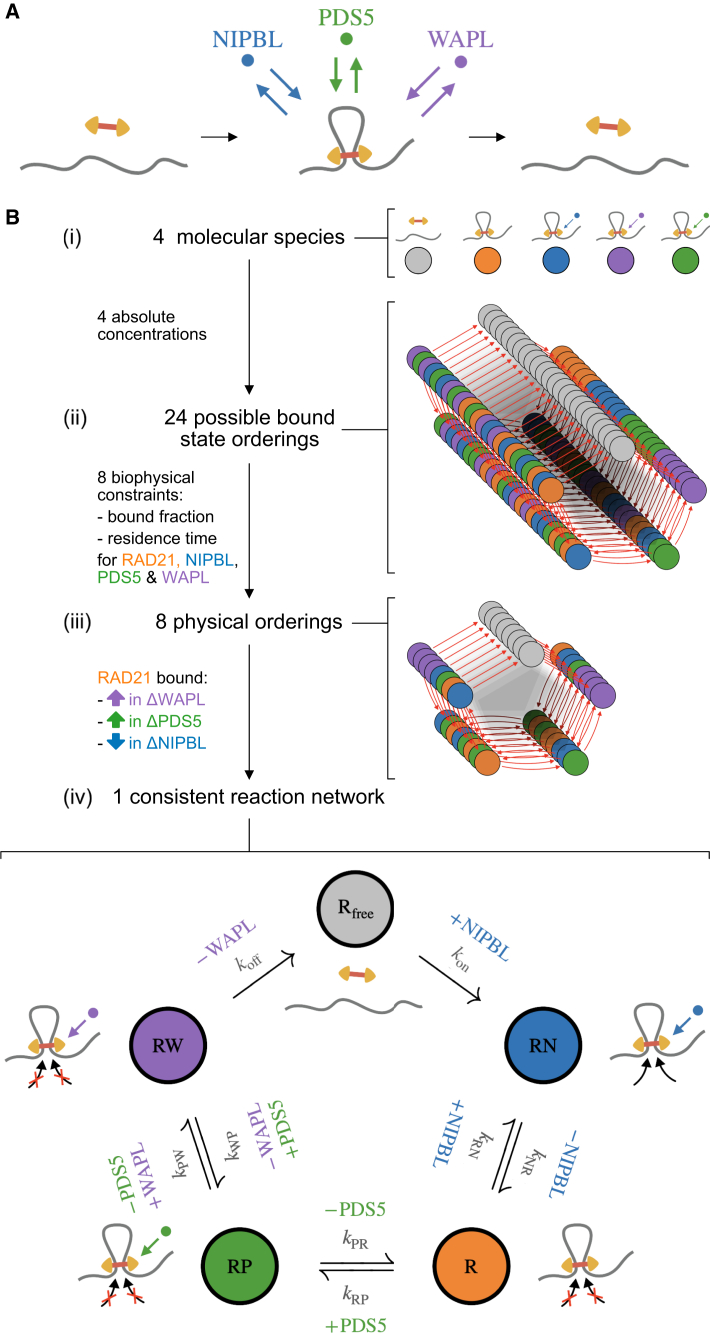
Determining the biochemical-reaction network of interphase cohesin (A) The cohesin complex loads, is reversibly bound by regulator proteins at short (∼1 min) timescales, and unloads at longer (∼10 min) timescales. (B) Pruning procedure to obtain a minimal biochemical network model describing the interplay of the regulatory proteins NIPBL, PDS5, and WAPL. (i) Modeling these three regulators along with the core complex (using RAD21 as its proxy) yields one unloaded and four loaded states. (ii) Pruning begins by considering all possible sequences (24) of regulator exchange on loaded cohesin complexes. (iii) After constraining the total nuclear abundance of each protein based on mass spectrometry and fluorescence correlation spectroscopy (FCS measurements in HeLa cells, only eight reaction networks were physically compatible with the experimental chromatin-bound fractions and residence times of RAD21, NIPBL, PDS5, and WAPL as estimated by FRAP in HeLa (see STAR Methods). (iv) Further using the fact that chromatin-associated cohesin increases for ΔWAPL and ΔPDS5 but decreases in ΔNIPBL after RNAi depletion^18^ yields a single cyclic reaction network consistent with *in vivo* observations. See also Figures S1–S3.

We next considered models describing cohesin biochemistry as a reaction cycle with irreversible loading and unloading transitions. For these reaction networks, loading of the core complex onto chromatin occurs via a one-way, irreversible, transition— potentially concomitant with the co-binding of a regulatory protein. After loading, additional cohesin regulators may reversibly bind and unbind the loaded core complex until its eventual irreversible unloading (Figure 1A). Such cyclic reaction networks confer distinct roles upon regulatory proteins based on their position within the cycle: the first co-binding factor acts as the primary loader, while the last co-binding factor acts as the primary unloader. To systematically consider the potential functions of each regulator, we determined the transition rates for each of 24 possible reaction cycles using biophysical data (Figure 1B). Remarkably, applying our two-stage pruning procedure uncovered a unique reaction cycle consistent with available data (Figure 1B). Given potential uncertainties in experimental quantifications, we further assayed the stability of this pruning protocol to changes in the abundance, chromatin-bound fraction, and residence time of cohesin and its regulators (Figure S2). This analysis revealed that the same cyclic reaction network remains the only viable five-state model over a large swathe of experimental parameter space, with the most stringent constraint stemming from the fact that the total amount of bound regulators may not exceed the overall loaded cohesin population owing to our assumption of strict regulator exchange. Thus, this cohesin biochemical network is generally robust to variations in the set of input measurements used for parametrization (Table 1).

Based on the order of states in the reaction network we obtained, NIPBL acts as the primary loader and WAPL as the primary unloader. PDS5 is required to recruit WAPL, but plays no direct role in unloading per se and also competes with NIPBL for association with the “bare” loaded cohesin (*R*) state. To understand how this unique network emerges from our imposed set of biochemical constraints, we tested whether reaction networks with modified topologies could also be reconciled with experimental data (Figure S3). We first considered a model where, instead of being directly involved in loading, the NIPBL-bound state constitutes an excursion from the main reaction cycle (Figure S3A). For this topology, however, NIPBL depletion does not sufficiently lower the loaded fraction of cohesin to agree with experiments.^18^ The failure of this topology thus supports a direct role for NIPBL in cohesin loading. We next considered a PDS5 excursion model, where PDS5 reversibly binds the core complex but does not recruit WAPL (Figure S3B). With this topology, ΔPDS5 actually slightly lowered RAD21 cohesin residence time instead of increasing it as observed experimentally.^18^ The inconsistency of this topology thus argues that PDS5 helps recruit WAPL to promote cohesin unloading.

Together, these observations show our minimalistic five-state cohesin reaction network recapitulates biophysical data for RAD21, NIPBL, PDS5, and WAPL and uniquely identifies their respective roles in the cohesin loading/unloading cycle.

### Steady-state turnover kinetics of cohesin regulators govern loop-extrusion activity

**Figure 2:**
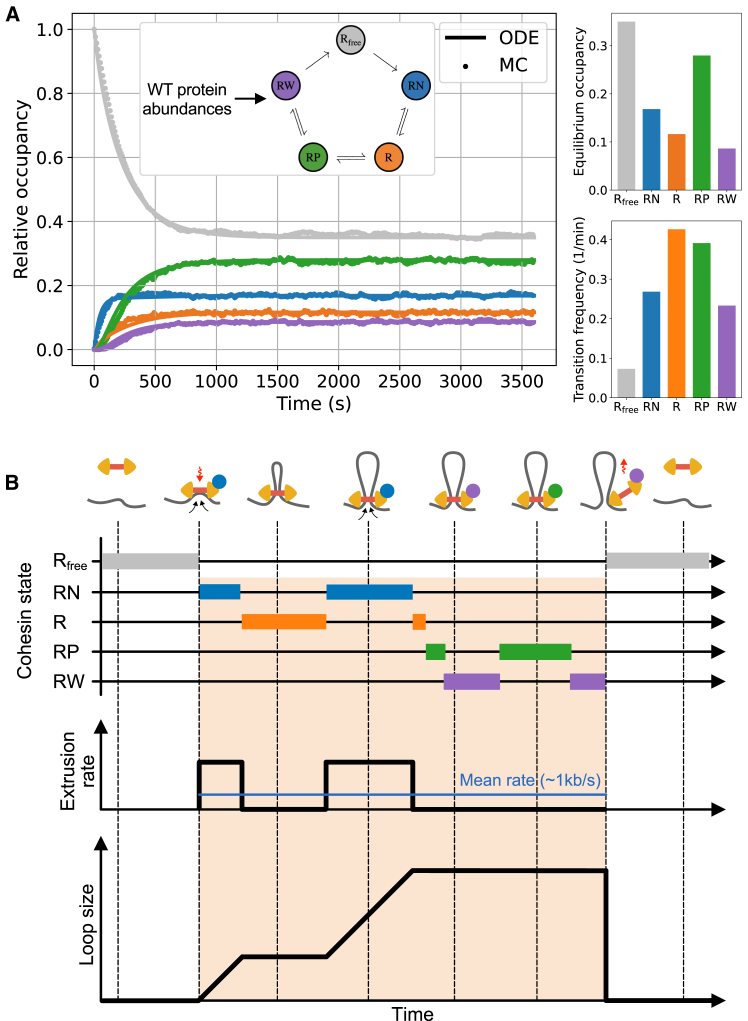
Biochemical-reaction network kinetics determine cohesin molecular properties (A) Model equilibration dynamics, as computed by integration of the mass-action ordinary differential equation (ODE; solid) or by a discrete-time kinetic Monte Carlo (MC; dashed) approach, starting from a fully unloaded cohesin population (*R*_*free*_ = 1; see STAR Methods). Right, top: equilibrium occupancies, normalized to the total cohesin nuclear content. Right, bottom: state transition frequencies, defined as the average number of transitions into each state per cohesin loading window divided by the cohesin residence time (as in Barth et al.^40^). (B) Example kymograph predicted by the bursty extrusion model. The mean translocation rate is controlled by the fraction of time spent by individual extruders in the active NIPBL-bound (RN) state (blue) while loaded onto chromatin and approaches 1 kb/s in WT HeLa cells^6^^,^^7^ (STAR Methods; Figure S4 and Video S1).

We next determined how the cohesin reaction network maps protein abundances to loop-extrusion kinetics. Based on the structure of our mass-action kinetic equations, we formally show in Methods S1, Existence and uniqueness of steady state that the chemical-reaction network in Figure 1B admits only a single equilibrium state, which is uniquely determined by the nuclear abundances of cohesin regulators.^39^ Indeed, starting from a fully unloaded cohesin population, our model rapidly reached a steady state characterized by a dynamic exchange of regulators (Figure 2A). Extruders are loaded onto chromatin upon transitioning from the free to NIPBL-bound state and unload upon transition from the WAPL-bound to the free state (Video M1). While loaded, each cohesin extruder transitions through a stochastic sequence of states concomitant with the reversible association of each cohesin regulator with the core complex (Figure 2B). Since NIPBL is required for ATP hydrolysis by the cohesin complex,^6^^,^^7^ we assumed that extrusion occurs only in the NIPBL-bound state (*RN*). As a result, the translocation kinetics predicted by the model alternate between periods of loop growth and stasis before the complex is finally unloaded (Figure 2B). We quantified this discontinuous extrusion behavior in terms of a mean translocation rate averaged over the entire lifetime of the loaded state, and henceforth referred to this cohesin description as the bursty extrusion model (Figure 2B).

**Figure 3:**
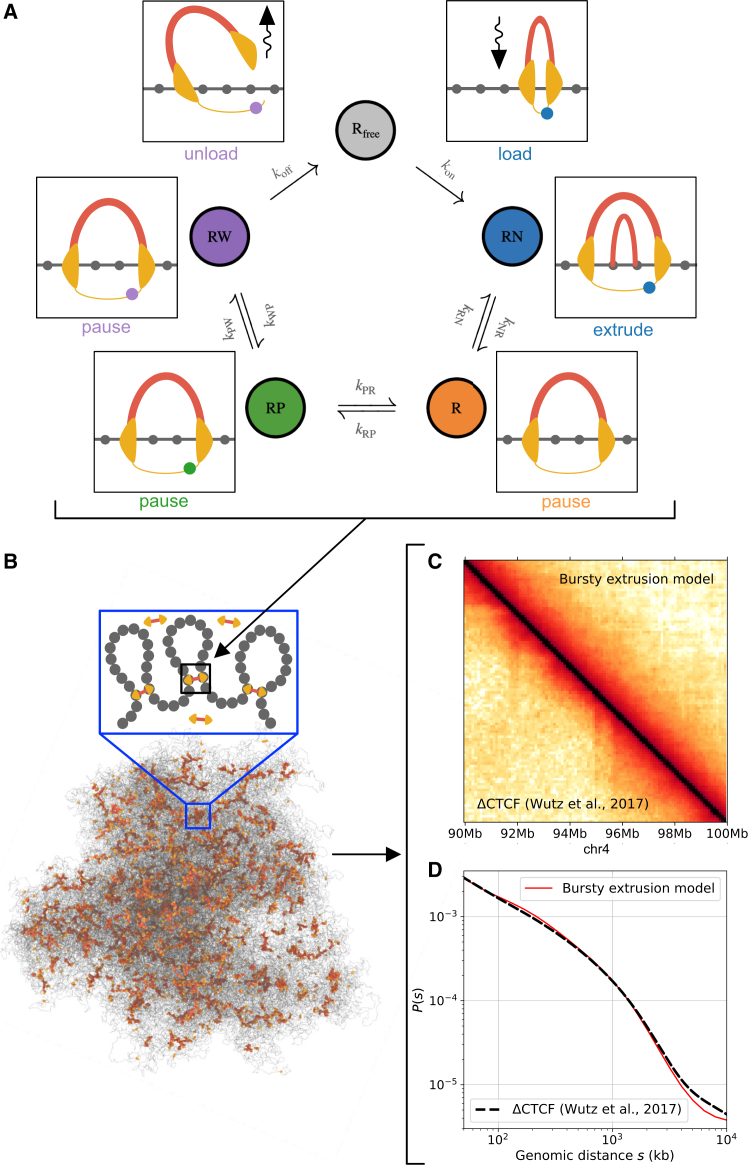
Coupling bursty extrusion model with polymer simulations yields quantitative predictions of 3D genome folding (A–C) Schematic representation of the coupled simulation workflow. Extrusion dynamics are computed based on a lattice-based MC scheme (A) and used as input for 3D MD simulations (B), from which *in silico* contact maps (C) or microscopy images may be generated (STAR Methods). Transition rates between states in the lattice simulations are parametrized using the bursty extrusion model (Table S1). Extrusion updates occur only in the NIPBL-bound (RN) state. Unloading happens upon transitioning between the WAPL-bound (RW) and free states, and loading happens upon transitioning between the free and NIPBL-bound states (Figure 2). (D) Contact frequency versus distance scaling curves, *P(s)*, either predicted by the bursty extrusion model (red) or obtained from experimental Hi-C in CTCF-depleted HeLa cells (dashed black^18^). See also Figures S5–S7 and Video S1.

Video S1. Exemplary loading cycle of an isolated cohesin extruder in WT HeLa conditions The cycle is as predicted by the bursty extrusion model, related to Figures 2 and 3.

Thus, the bursty extrusion model provides a natural representation of cohesin as a multi-state molecular motor, whose activity is controlled by dynamic and reversible association with NIPBL. This prediction is corroborated by recent single-molecule tracking experiments, which revealed that individual loaded cohesins may alternate between active (extruding) and passive phases through transitions that strongly correlate with NIPBL-binding/unbinding events.^40^ Cohesin was further found to transition into the active state at a rate of ∼1 per minute,^40^ consistent with the ∼0.3 transitions per minute into the RN state predicted by the bursty extrusion model (Figure 2A). Additionally, the model predicts that only ∼17% of all loaded and unloaded interphase cohesins are actively extruding at any point in time, quite close to *in vitro* measurements in standard buffer conditions (∼18%^41^). Agreement with these numbers indicates that extended periods of cohesin inactivity while loaded on chromatin are a key feature of the bursty extrusion model.

### Coupling the bursty extrusion model with polymer simulations predicts 3D genome folding

To compare predictions of the bursty extrusion model with experimental Hi-C, we used our cohesin reaction network as an input for polymer simulations of chromatin. As for previous approaches,^42^^,^^43^ we coupled a 1D lattice model of cohesin translocation, which tracks cohesin positions over time along the genome, with coarse-grained molecular dynamics (MD) simulations of a generic chromosome, which track chromatin and extruder positions in 3D (STAR Methods). In the lattice model, cohesins stochastically load, unload, and extrude chromatin. However, we modified cohesin activity in the lattice model to directly depend on the states and transition rates extracted from our bursty extrusion model (Table S1). To simulate stochastic transitions between states for individual cohesin complexes, we used a discrete-time kinetic Monte Carlo approach (Figure 2A). As hypothesized for condensin-condensin interactions,^44^ we further assumed that cohesin-cohesin encounters along the lattice lead to collisions without bypass. Thus, in the chromatin-loaded states lacking NIPBL (*R*,*RP*,*RW*), cohesin remains immobile yet still blocks translocation by active extruders. For parsimony, we considered that transition rates between states are homogeneous across all genomic positions.

Using the bursty extrusion model, we simulated the lattice translocation dynamics of ∼6,700 cohesin extruders on 500 Mb of chromatin at 2.5-kb resolution (STAR Methods), representing 50 copies of a 10-Mb region of the genome. As 35% of extruders are dissociated at steady state, simulations have a mean density of ∼8.7 loaded cohesins per Mb, consistent with the numbers reported in G1 HeLa cells. To parameterize the average extrusion rate in simulations, which depends on the fraction of time a chromatin-associated extruder is in the NIPBL-bound state (Figure 2B), we used the average rate reported *in vitro* for human cohesin on naked DNA (*v* = 1 kb/s^6^). We then obtained and quantified *in silico* Hi-C data by generating an ensemble of 5,000 chromatin conformations, recording chromatin contacts, and computing the corresponding contact-versus-distance (*P*(*s*)) scaling curve (Figure 2B). We then compared simulated and experimental *P*(*s*) obtained from a 10-Mb region of chromosome 4 (chr4:90-100Mb) without evident translocations. The simulated *P*(*s*) curve quantitatively matched measurements in HeLa cells after CTCF depletion (ΔCTCF; *R*^2^ > 0.99; Figure 3D), which provided an optimal reference for comparison as the current model did not include extrusion barriers. If we instead treated extrusion rate as an adjustable parameter, we found optimal agreement at 850 bp/s, similarly consistent with the range of *in vitro* estimates (0.5–1 kb/s^6^^,^^7^; Figure S5).

Interestingly, this optimal average extrusion rate, combined with the inference that only a subset (27%) of loaded cohesins are active, implies that the extrusion rate of NIPBL-bound cohesin approaches ∼3,150 bp/s. While such rapid kinetics have been infrequently observed in loop-extrusion traces *in vitro*,^6^^,^^7^ they are remarkably consistent with the rates of ∼2,700 bp/s recently measured during active cohesin extrusion windows based on ultra-resolution live imaging *in vivo*^45^ and inferred for condensin in mitosis.^44^ Thus, another noteworthy prediction of the bursty extrusion model is that cellular cohesin achieves instantaneous chromatin extrusion rates substantially higher than those reported *in vitro*.

The staggered translocation kinetics from the bursty extrusion model had a small yet discernable impact on the simulated *P(s)* curve when compared with previous one- and two-state models of extrusion. These respectively assume a constant translocation rate with or without immediate reassociation upon unloading^4^^,^^46^^,^^47^ and displayed slightly worse agreement with experimental data at the same average extrusion rate (Figure S6A). Nonetheless, the relative similarity in the *P(s)* predictions of the different models likely stems from the fact that Hi-C is a population-level analysis, implying that this metric may not fully reflect the additional heterogeneity in single-cohesin properties provided by the bursty extrusion model (Figures S6B and S6E; Video M1). In contrast, extruder heterogeneity is required to reproduce the dispersion observed *in vitro* for single-molecule properties like the extrusion rate^6^^,^^7^ alongside their association with cohesin biochemical state—as recently reported for NIPBL-binding events.^40^

To more broadly understand how heterogeneous extrusion impacts genome organization, we considered how two modifications alter predictions for simulated *P(s)*: (1) CTCF barriers; (2) diffusion of cohesins along chromatin. To assay the impact of CTCF, we coupled the bursty extrusion model with a dynamic model of CTCF boundaries.^48^ We focused on the average region-wide impacts of CTCF barriers rather than locus-specific features such as topologically associating domains (TADs) or dots, as current experiments do not ascertain site-specific CTCF-bound times.^49^ Using CTCF nuclear copy number, chromatin residence time, and bound-fraction measurements reported in live HeLa cells,^36^^,^^37^^,^^50^ we found that the addition of CTCF only mildly affected the simulated *P*(*s*) curves and chiefly led to a limited reduction in chromatin contacts in the genomic span (100 kb: 4,000 kb) (Figure S7A). Interestingly, addition of CTCF to the bursty extrusion model provided slightly better agreement than CTCF added to a continuous (two-state) extrusion model (Figure S7B). We attribute the improved accuracy of the bursty extrusion model to an effectively increased blocking efficiency of CTCF barriers. In particular, bursty extruders can only attempt to bypass a CTCF barrier when they are active and NIPBL bound, rather than throughout their lifetime.^48^^,^^50^

We next assayed the impact of stochastic, one-dimensional diffusion on loaded cohesins via an NIPBL- and ATP-independent process, as reported *in vitro* on naked DNA^6^^,^^7^ and Xenopus extract.^51^ Since mechanistic details remain uncertain, we tested two possibilities for how this occurs: either with both legs sliding in tandem or independently. In general, both types of diffusive motion generally yielded only limited effects on the simulated *P*(*s*) curves. If legs slid independently, experimental Hi-C data can be potentially reconciled with a cohesin diffusion rates up to *D* ≈ 0.4 kb^2^/s—consistent with the *in vitro* estimate *D* ≈ 0.5 kb^2^/s^40^ (Figure S7C). However, if both legs slid in tandem, they did not produce any measurable changes in contact-versus-distance curves at the diffusion rates considered (Figure S7D). Given the overall limited impacts of both cohesin diffusion and CTCF barriers on genome-wide organization as captured by *P(s)*, we thus returned our focus to the unmodified bursty extrusion model for the rest of the paper.

Collectively, our coupled bursty extrusion and polymer models quantitatively predict extrusion kinetics and resulting 3D genome structure based solely on biophysical measurements without relying on any input from Hi-C data.

### Bursty extrusion model maps cohesin-regulator abundance to translocation dynamics

**Figure 4:**
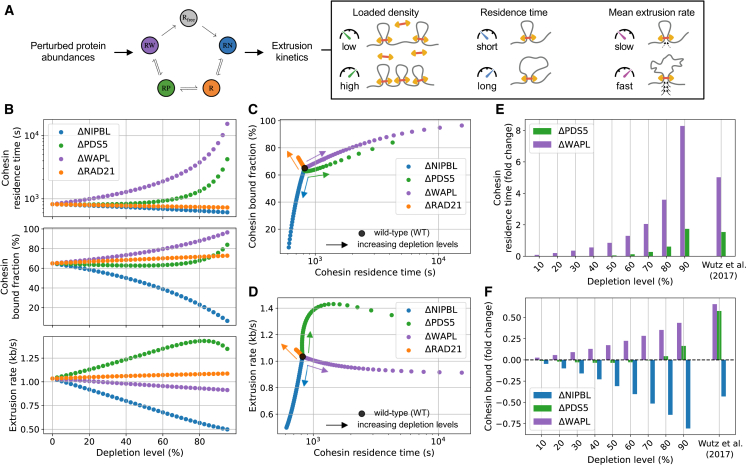
Bursty extrusion model reveals non-linear relationship between regulator abundance and cohesin motor properties (A) Simulation workflow for *in silico* perturbation experiments. Altered protein abundances are provided as input to the bursty extrusion chemical-reaction network using kinetic rates computed previously with WT measurements (cf. Figure 2A). Perturbed extrusion properties may then be extracted directly from the new equilibrium state of the network (cf. Figure 2B). (B) Predicted change in cohesin residence time (top), chromatin-loaded fraction (center), and extrusion rate (bottom) as a function of depletion level for different cohesin subunits in simulations in 2.5% increments. (C) Residence time versus loaded fraction as a function of depletion level from the WT values (black circle). Data are as in (B), and arrows show direction of increasing depletion. (D) Extrusion rate versus residence time. Symbols and data are as in (C). (E) Change in cohesin residence time relative to WT as a function of depletion level in simulations (from 10% to 90% in 10% increments) and experimental data.^18^ (F) Same as in (E) for the loaded cohesin fraction. See also Figures S8 and S9.

The bursty extrusion model derives from an analytical one-to-one mapping from experimental measurements in wild-type (WT) HeLa cells to biochemical transition rates between cohesin states. In the framework of mass-action kinetics, these rates subsume the dependence of chemical kinetics on external reaction conditions but do not depend on regulator abundance. Thus, the bursty extrusion model can directly predict how changes in protein levels influence loop translocation kinetics by altering their abundances and computing the resulting network dynamics, keeping the analytical transition rates fixed (Figure 4A; cf. Methods S1, Rate mapping procedure).

Since the bursty extrusion model explicitly describes the association/dissociation process of each regulator, we were thus able to independently assess the effect of their individual or combinatorial depletions on cohesin behavior. We first performed *in silico* depletions by systematically varying abundance of each individual regulator from WT levels down to 95% depletion, used altered abundances as input to the bursty extrusion reaction network, and computed predicted extrusion properties after depletion from the equilibrium state of the network (Figure 4A). The bursty extrusion model predicted that such depletions could produce non-linear impacts on multiple extrusion properties. We found (1) ΔNIPBL increased extrusion rate and lowered the loaded fraction, (2) ΔWAPL increased loaded fraction and residence time, (3) ΔPDS5 changed all three extrusion properties, and (4) ΔRAD21 chiefly lowered the number of cohesins on chromatin (Figures 4B–4D). Consistent with the ΔNIPBL prediction for a limited decrease in residence time, a minimal reduction in loop lifetimes was reported *in vitro* upon lowering the ratio of NIPBL to RAD21.^40^ The lower extrusion rate predicted in ΔNIPBL mirrors experimental observations of lower ATP hydrolysis rates by cohesin when the availability of NIPBL is reduced.^6^^,^^7^^,^^26^^,^^52^ Conversely, the higher extrusion rate predicted for ΔPDS5 resulted from a higher frequency of re-binding NIPBL from the bare loaded cohesin state, leading to an upturn in the NIPBL-bound cohesin population (RN), which similarly aligns with experimental observations.^26^ In our model, an increased extrusion rate is eventually compensated by an increased loaded cohesin fraction at higher depletion levels of PDS5 (Figure 4B), which also lowers the availability of NIPBL per loaded cohesin. These competing effects led to a non-monotonic dependence of extrusion rate on PDS5 levels, characterized by an initial increase followed by a moderate decrease at very high PDS5 depletion levels (Figure 4B).

To compare with experiments, we focused on the case of 90% depletion of the different regulators, corresponding to estimated depletion levels after RNAi.^18^ At this depletion level, cohesin residence time was predicted to increase 8-fold after ΔWAPL or 2-fold after ΔPDS5 (Figure 4E), in close agreement with experimental FRAP after either ΔWAPL or ΔPDS5A + B by RNAi.^18^ Importantly, cohesin residence times after regulator depletions were not used to fit the bursty extrusion model and thus provided orthogonal model validation. We next considered predictions for the chromatin-loaded fraction of cohesin, again after 90% depletion. The bursty extrusion model predicted a greater increase after ΔWAPL than ΔPDS5 (∼40% vs. ∼20%; Figure 4F). These values are ordered identically but are slightly lower than experimental estimates (∼65% vs. ∼55%, respectively^18^). The discrepancy for the loaded fractions can be alleviated by considering an alternative model where WAPL and PDS5 simultaneously co-bind RAD21^23^ and are jointly required for cohesin unloading. However, predicted increases in residence times for this strict co-binding model substantially overestimated those observed in experimental data (Figure S8). In contrast to the increased loaded fraction after ΔWAPL or ΔPDS5, the bursty extrusion model predicted that ΔNIPBL would decrease the loaded cohesin fraction, consistent with the 45% reduction reported experimentally.^18^ Since RNAi efficiency was not reported for ΔNIPBL, we considered a range of depletion levels *in silico* and found the best agreement with experiments occurred at 60% depletion (Figure 4F).

The bursty extrusion model also predicts the residence time and bound fraction of NIPBL, PDS5, and WAPL after depletion of any cohesin regulator. For instance, it suggests that ΔWAPL increases the chromatin-associated fraction of NIPBL but minimally alters its residence time (Table S2)—in agreement with FRAP experiments after perturbation *in vivo*.^28^ Similarly, it predicts the chromatin residence time of NIPBL is largely independent of its expression level, consistent with single-molecule observations of cohesin extrusion *in vitro*.^40^ This feature of our model stems from the fact that the release of NIPBL via the RN → R pathway does not involve the direct recruitment of other regulators. Since NIPBL association governs cohesin translocation activity, this in turn implies that the mean rate of cohesin extrusion is modulated chiefly by the frequency of NIPBL-binding events (Figure S4) rather than the duration of active (RN) translocation windows, which also mirrors recent *in vitro* measurements of cohesin extrusion traces.^40^ Agreement with these features of experimental data highlight how our model captures the coupled dynamics of cohesin regulators, in addition to their effects on cohesin extrusion.

To explore the synergistic or antagonistic roles of cohesin regulators, we further considered some of their combined depletions *in silico*. The bursty extrusion model predicted that the stoichiometric co-depletion of WAPL and NIPBL would lead to cohesin loop patterns quantitatively similar to those observed in unperturbed cells, albeit with much longer residence times and slower extrusion rates for individual extruders (Figures S9A–S9D). Conversely, the co-depletion of WAPL and RAD21 failed to recover the WT phenotype, as the increased cohesin residence time induced by ΔWAPL is in this case not offset by a reduction in the rate of extrusion—resulting in a considerable increase in loop sizes (Figures S9E and S9F). This predicted WAPL/NIPBL compensation yields a mechanistic explanation for the reported rescue of genome folding following combined depletion of WAPL and NIPBL in Hap1^21^ and HCT116 cells^53^ and illustrates how the interplay between regulators dictates the fine balance between cohesin loading, unloading, and extrusion activity.

Together, the agreement between *in silico* depletions and experimental observations shows the power of the bursty extrusion model to predict how cohesin translocation dynamics are modulated by variations in regulator abundance—an ability so far inaccessible to previous models of cohesin loop extrusion.

### Bursty extrusion model predicts consequences of protein depletions on chromosome morphology and genome conformation

**Figure 5:**
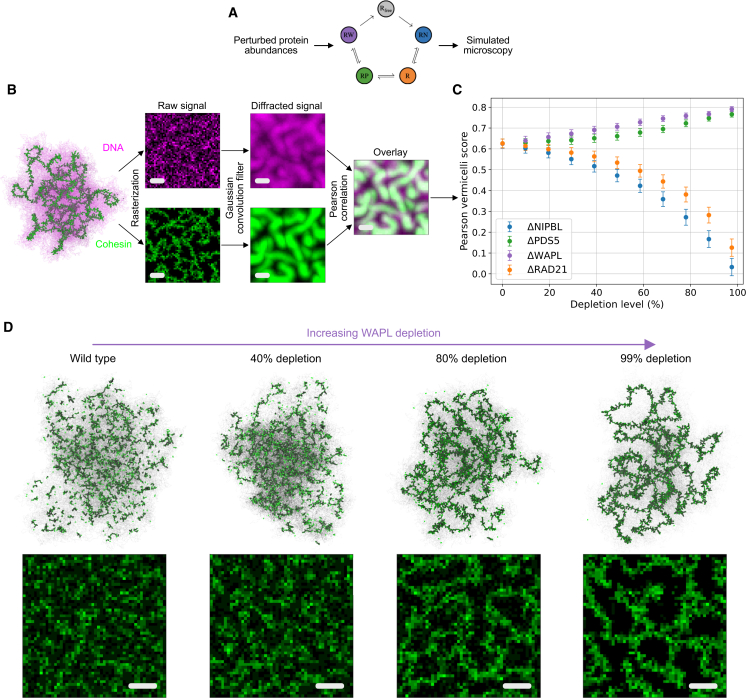
Bursty extrusion model maps protein abundance to 3D chromosome morphology (A) Predictions of cohesin extrusion kinetics at WT or altered protein levels (cf. Figure 3) can be used as input for polymer simulations to quantitatively predict chromosome dynamics and spatial organization. (B) Vermicelli score computation workflow. Cohesin (green) and DNA (magenta) spatial positions are separately tagged and binned into discrete 3D voxels. The two resulting rasters are subsequently run through a Gaussian convolution filter to mimic the effects of optical diffraction. The vermicelli score is then defined as the Pearson correlation of the processed cohesin and DNA signal (STAR Methods). Scale: 1 μm. (C) Vermicelli scores as a function of simulated depletion level for indicated factors. Depletion of WAPL and PDS5 promote vermicelli formation; NIPBL and RAD21 depletion do not. Error bars were computed as the standard error of the mean across 5,000 MD frames obtained from 5 independent simulations. (D) Top: polymer conformations, showing extruder positions (green). Bottom: simulated microscopy of RAD21 localization. Both are displayed as a function of WAPL depletion level showing the emergence of vermicelli. See also Figures S10 and S11.

To investigate how altered loop-extrusion properties translate into changes in 3D genome folding, we repeated our *in silico* mutant analysis using polymer simulations coupled to the bursty extrusion model and computed observables that could be compared with either experimental Hi-C or microscopy. We first explored the effects of regulator depletions on 3D chromosome morphology by performing *in silico* microscopy for both cohesin and chromatin (Figure 5A). We extracted the spatial positions of cohesins and chromatin from individual conformations, rasterized into a 3D voxel grid, and performed convolution with a Gaussian kernel (Figure 5B). Visually, as WAPL depletion increases, a granular cohesin backbone emerges, which results from an accumulation of collided extruders along the chromatin fiber (Figures 5B–5D). We quantified this using a “vermicelli score,” defined as the Pearson correlation coefficient of the resulting simulated cohesin and DNA fluorescence signals (Figures 5B and 5C). Unlike previous metrics based solely on cohesin fluorescence intensity,^17^ this measure has the advantage of being normalized by construction, with a value of 1 indicating complete overlap of cohesin and DNA foci—reflecting ideal vermicelli condensation^54^—and a value of 0 denoting full decorrelation of the diffuse cohesin and DNA signals.

This vermicelli score increases with ΔWAPL or ΔPDS5 but decreases in ΔRAD21 or ΔNIPBL—whose depletion did not prompt vermicelli patterns (Figures 5C; S10). Model predictions agree with experimental *in situ* fluorescence microscopy, which reported vermicelli upon ΔWAPL or ΔPDS5AB contrasted by a loss of the chromatin-associated cohesin in ΔNIPBL.^18^ Auxiliary measures of vermicelli formation, namely the coverage by loops or the collided fraction of loop extruders, largely mirrored the microscopy-based Pearson vermicelli score (Figures S11A and S11B). In addition to these intuitive metrics, we also quantified vermicelli formation via a percolation score, computed as the fraction of loaded cohesins comprising the largest cluster of collided extruders. Visually, this score appears to better capture the extent of vermicelli formation and displays a sharp increase upon depletion of WAPL and PDS5 beyond 80% (Figures S10 and S11C). We note, however, that these alternative vermicelli metrics rely on simultaneous knowledge of all extruder leg positions along the chromosome and hence are not currently accessible to direct experimental measurements.

**Figure 6:**
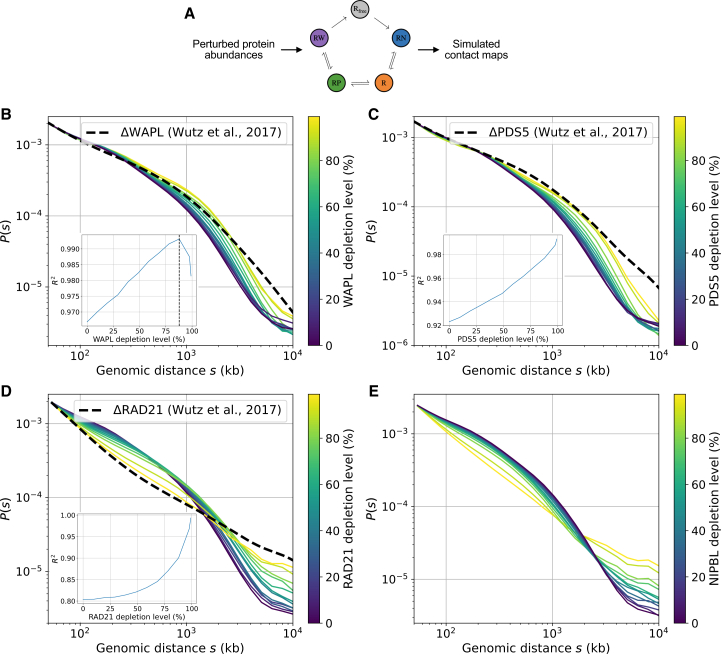
Bursty extrusion model quantitatively relates cellular stoichiometry and genome-wide contact patterns (A) Statistical ensembles of polymer conformations simulated at fixed nuclear protein levels as in Figure 5 can be aggregated to yield population-averaged contact (Hi-C) maps in both WT cells and at arbitrary depletion conditions. (B) Contact frequency versus distance curves for simulated ΔWAPL (colored by depletion level), with experimental RNAi depletion (dashed line) from Wutz et al.^18^ Inset shows best fit is reached at 88% depletion. (C) Same as (B), for simulated ΔPDS5 and experimental auxin-induced degron (dashed line) from Wutz et al.^18^ Inset shows best fit is approached at the highest depletion levels considered in simulations (99%). (D and E) Same as (C) for simulated ΔRAD21 (D) and ΔNIPBL (E). Note that corresponding experimental measurements in NIPBL-depleted HeLa cells are, to our knowledge, currently lacking.

To further assay the effects of altered loop extrusion on 3D genome conformations, we extracted *P*(*s*) from *in silico* and *in vivo* Hi-C (Figure 6A). We observed that ΔWAPL leads to a rightwards shift of the characteristic “shoulder” in these curves associated with loop extrusion (Figure 6B).^3^^,^^55^ By computing the goodness-of-fit (*R*^2^; STAR Methods), the experimental data were best reproduced by WAPL depletion levels of around 85% (Figure 6B), consistent with the RNAi efficiency estimated experimentally.^18^ ΔPDS5 displayed a similar shift in the shoulder, again congruent with experimental data, albeit without evidence for a clearly best-fitting degradation level (Figure 6C). Conversely, ΔRAD21 had an entirely different impact on the *P*(*s*) in both simulation and experiments and instead resulted in the gradual disappearance of the shoulder (Figure 6D). In this case, optimal agreement occurred at RAD21 depletion levels greater than 99%, consistent with its highly efficient removal via auxin-induced degradation.^18^ Simulated ΔNIPBL also led to a gradual disappearance of the shoulder, but the corresponding experimental Hi-C data in NIPBL-depleted HeLa cells have, to our knowledge, yet to be reported (Figure 6E).

Collectively, our *in silico* microscopy and Hi-C results indicate that the minimalistic description of cohesin biochemistry provided by the bursty extrusion model quantitatively captures how the levels of cohesin and its regulators jointly determine genome folding.

## Discussion

In summary, we present a quantitative minimal model of the cohesin biochemistry underlying interphase loop extrusion, parametrized solely by biophysical data on the dynamics of cohesin and its regulators. We find that a single reaction network, characterized by a cycle involving five cohesin states, can be fully reconciled with protein abundances, FRAP, and cohesin immunoprecipitation assays after individual regulator depletions in HeLa cells. Combining this multi-state, bursty extrusion model with polymer simulations enables direct predictions of 3D genome folding from nuclear protein levels, and shows how the transient binding of cohesin regulators may modulate loop-extrusion kinetics across multiple time- and length scales.

In stark contrast with previous models of interphase cohesin, the bursty extrusion model predicts that cohesin translocation does not proceed continuously but rather is characterized by periods of immobility interspersed with windows of activity resulting from transient NIPBL binding. These observations closely mirror single-molecule experiments displaying staggered, NIPBL-associated extrusion kinetics *in vitro*.^40^ However, the bursty extrusion model additionally predicts that translocation rates *in vivo* may reach up to ∼3 kb/s. This predicted instantaneous rate is substantially faster than those reported by single-molecule experiments for cohesin *in vitro*^6^^,^^7^ but is consistent with recent independent measurements by ultra-resolution chromatin tracking *in vivo*^45^—and is also intriguingly close to the values estimated for condensin in mitosis.^44^^,^^56^ Together, these lines of evidence suggest that *in vitro* assays currently underestimate the physiological rates of cohesin translocation. Such discrepancies could stem from the additional stretching forces imposed by the microfluidic setup employed in these experiments,^6^^,^^7^ which could serve to lower the observed translocation rates. Alternatively, underestimations could reflect the fact that *in vitro* measurements are performed on naked DNA; the chromatin template may boost the effective extrusion rate per base pair if a given step size in nanometers captures more DNA when wrapped around nucleosomes.^3^ This hypothesis suggests that modifications of chromatin would be effective ways to modulate the locus-specific kinetics of loop extrusion.

Furthermore, the bursty extrusion model concisely articulates knowledge of cohesin biochemistry and the respective functions of cohesin regulators. The loading action of NIPBL^57^ and the unloading function of WAPL^34^ have both been long hypothesized based on sister-chromatid cohesion phenotypes. The contributions of PDS5 appear more multifaceted, and have been proposed to include both the promotion of cohesin unloading in cooperation with WAPL^58^ as well as competition with NIPBL for the same RAD21-binding site.^25^ These functions naturally emerge from the bursty extrusion model, whose network topology suggests that PDS5 not only plays a role in the recruitment of WAPL but also hinders the re-binding of NIPBL to loaded cohesin. Indeed, the molecular picture suggested by the bursty extrusion model is consistent with recent inferences of a role for PDS5 in setting the average extrusion rate of cohesin complexes.^59^^,^^60^ Additionally, our modeling approach enabled us to rule out large swaths of incompatible alternate topologies (Figures S1 and S3) and provide mathematical support for the concept that distinct loading and unloading pathways operate for loop extrusion as well as sister chromatid cohesion.

Our framework also yields mechanistic insight into various biophysical and genomic observations. First, the model demonstrates how the relatively rapid exchange of cohesin regulators can regulate loop-extrusion dynamics over much longer timescales. For instance, it quantitatively reconciles the short residence time of WAPL (∼1 min^22^) with the dramatic increase in cohesin residence time induced by its depletion (>1 h^17^) as well as the associated emergence of the vermicelli morphology at the chromosome-wide level.^17^^,^^18^ Furthermore, our model argues that contrasting conclusions drawn either from RNAi or more efficient auxin-induced degradation^61^ could result simply from the different degrees of depletion achieved by the different experimental protocols, combined with the predicted non-linear relationships between protein levels and extrusion properties.

Beyond the kinetics of the core complex, the bursty extrusion model highlights informative new experiments based on monitoring the dynamics of regulators in various conditions. Separate measurements for PDS5A and PDS5B binding kinetics could enable us to refine model predictions and resolve the moderate discrepancies for predicted contact-versus-distance scaling in simulated ΔPDS5. Existing experiments do not exclude a model where PDS5 and WAPL simultaneously bind RAD21 and are jointly required for cohesin unloading.^23^^,^^62^ Our modeling indicates, however, that measurements of the PDS5 residence time before and after WAPL depletion could support or exclude this possibility: if the PDS5 residence time does not substantially change in ΔWAPL cells, this would support a model where cohesin is unloaded via a co-bound PDS5/WAPL state (Figure S8; Table S3). Conversely, an increase of the PDS5 residence time upon WAPL depletion would suggest that the role of PDS5 primarily lies in the recruitment of WAPL, which in turn acts as the standalone primary cohesin unloader (Table S2). Such measurements of regulator kinetics in perturbed cells are currently largely lacking but would now provide important benchmarks due to the powerful predictive ability of biochemical-reaction network models.

By virtue of its minimalist approach and increased biophysical accuracy, the bursty extrusion model provides a platform to systematically explore the rules and biochemistry governing cohesin motion in future work. More complex models could incorporate other known regulators of cohesin such as SA1/2, SCC4, and Sororin,^12^ as well as the role of SMC3 acetylation.^63^^,^^64^ Future work can also consider how the chemical state of cohesin impacts the barrier activity of CTCF, including via competition with WAPL^65^ or collaboration with PDS5.^66^ Accounting for these regulators will be crucial for future models of cohesin biochemistry throughout the cell cycle, including for the establishment of sister chromatid cohesion.^67^

Furthermore, the implementation and evaluation of more complex network topologies with additional transitions will also be of future interest, even for set of currently modeled regulators. For example, while the failure of the alternative NIPBL excursion topology argues for the requirement of NIPBL in loading into the extrusion cycle (Figure S3A), it does not rule out models with transient reversible associations of cohesin with chromatin prior to NIPBL-associated loading.^21^^,^^22^ Consideration of this alternative topology with additional transition rates and states could refine model predictions by increasing the background population of chromatin-associated cohesins at high levels of NIPBL depletion. Moreover, topologies with additional unloading pathways would be useful to evaluate the importance WAPL-independent cohesin dissociation.^68^ Consideration of such pathways could refine model predictions by imposing a finite cohesin residence time on chromatin even upon full WAPL knockout.

Since the nanoscale details of how motor conformational changes result in loop extrusion remain uncertain,^1^ we assumed that individual extruders symmetrically reel in chromatin. Many alternatives and elaborations are likely, including asymmetric extrusion with switching,^40^^,^^69^ a dependence of the loop-extrusion rate on local conformation,^70^ cohesin backtracking and bypassing,^71^ or capture of spatially proximal chromatin in *trans*.^72^ Future models will be required to consider locus-specific cohesin properties *in vivo*, such as targeted loading or the interplay between cohesin and the transcription machinery,^31^ which could differentially modulate the extrusion rate in passive and actively transcribed regions.^73^ Alternatively, our framework for modeling cohesin regulators could be complemented with factors crucial for dissecting cohesin functionality *in vitro*. Indeed, previous models of SMC stepping dynamics^27^^,^^70^^,^^71^^,^^72^ could be naturally incorporated to describe the NIPBL-bound state. This would also provide a convenient way to account for how the cohesin stepping rate in the NIPBL-bound state varies as a function of mechanical tension on DNA or the concentration of ATP in solution.^27^

Despite the many possible elaborations for the rules of cohesin translocation, our bursty extruders with simple blocking collisions that operate uniformly across the genome produce contacts-versus-distance curves in excellent agreement with experimental Hi-C. Moreover, the inclusion of ATP-independent cohesin diffusion at rates suggested by experiments in simulations yielded only limited effects on contacts-versus-distance curves. In HeLa cells, our results thus argue that cohesin kinetics and collisions are the dominant factors determining interphase loop sizes, to which additional mechanisms potentially contribute as higher-order effects. Similar conclusions were recently reported for condensin-based loop extrusion in mitotic cells,^44^ which suggests that collision-based encounter rules between extruders of the same type could be evolutionarily conserved across SMC complexes.

To conclude, the bursty extrusion model establishes a minimalistic description of cohesin biochemistry capable of quantitatively capturing the roles of key cohesin regulators. It puts forth a molecular paradigm for interphase loop extrusion centered on cohesin as a multi-state motor. Our results highlight the ability of simple biophysical models to integrate data from multiple orthogonal modalities, including *in vitro* motor assays, quantitative *in vivo* measurements, genomics, and *in situ* immunofluorescence microscopy to build a holistic picture of chromosome organization. Altogether, our framework illuminates how cells can harness loop extrusion by fine-tuning regulator abundances across cell types and states, thus bridging the gap between our molecular and genome-scale understanding of chromosome organization.

### Limitations of the study

The bursty extrusion model as presented here does not explicitly account for several known cohesin regulators, including SA1/2, SCC4, Sororin, and the role of SMC3 acetylation. The current model is limited to HeLa cells, where sufficient biochemical data exist to infer model parameters from first principles. Extensions to other cell types will require the use of either additional experimental datasets or new approaches for determining model kinetics. The current model amalgamates PDS5A and PDS5B, as distinct FRAP measurements to differentially parameterize their effects were unavailable. The presented model considers that regulators bind mutually exclusively to the core complex and that regulatory proteins require the core complex to be chromatin associated. The model also currently assumes cohesin performs strictly symmetric loop extrusion with uniform kinetics across the genome and thus does not account for locus-specific effects such as targeted loading or transcription-dependent barriers. Similarly, CTCF barriers were incorporated based on their average genome-wide properties rather than site-specific kinetics due to an experimental lack of existing locus-specific CTCF-bound times and occupancy measurements. The proposed description of extrusion kinetics does not consider how potential cohesin-cohesin bypassing or *trans*-capture of spatially proximal chromatin would impact predictions. Finally, the current approach is limited to cohesin extrusion activity and does not address how regulator exchange might impact the establishment or maintenance of sister chromatid cohesion.

## Resource availability

### Lead contact

Requests for further information and resources should be directed to and will be fulfilled by the lead contact, Geoffrey Fudenberg (fudenber@usc.edu).

### Materials availability

This study did not generate new unique reagents.

### Data and code availability

• Simulation codes for biochemical kinetics calculations, lattice loop-extrusion simulations, MD computations, and associated data analyses are all publicly available as detailed in the key resources table.
• Simulation data will be provided upon request to the lead contact.

## Acknowledgments

We thank Elphège Nora, Anton Goloborodko, Erika Anderson, and Gordana Wutz for helpful discussions and detailed feedback; Max Imakaev for detailed code repository feedback; as well as of the Fudenberg research group for helpful conversations.

Funding was received from 10.13039/100000057National Institute of General Medical Sciences grant R35GM143116 (G.F.).

## Author contributions

M.M.C.T. performed analytical calculations, carried out numerical implementations, and conducted simulations. Both authors designed the project, analyzed data, and wrote the manuscript.

## Declaration of interests

The authors declare no competing interests.

## STAR★Methods

### Key resources table

**Table:** 

REAGENT or RESOURCE	SOURCE	IDENTIFIER
**Deposited data**		

Experimental Hi-C data for HeLa cells	Wutz et al.^18^	GEO: GSE102884

**Software and algorithms**		

Biochemical network model	Tortora et al.^74^	https://github.com/Fudenberg-Research-Group/multistate-extrusion-networks
Lattice loop extrusion codes	Tortora et al.^75^	https://github.com/Fudenberg-Research-Group/discrete-time-extrusion
polychrom-hoomd	Tortora et al.^76^	https://github.com/open2c/polychrom-hoomd
polykit	Tortora et al.^77^	https://github.com/open2c/polykit
cooler	Abdennur et al.^78^	https://github.com/open2c/cooler
cooltools	Abdennur et al.^79^	https://github.com/open2c/cooltools
pairtools	Open2C et al.^80^	https://github.com/open2c/pairtools
distiller	Goloborodko et al.^81^	https://github.com/open2c/distiller-nf

### Method details

#### Abundance & dynamics of cohesin & regulators

Determining the transition rates for our minimal model of interphase cohesin chemistry requires three quantities – namely, abundance, bound fraction, and residence times measured in unperturbed cells – for each regulator considered. For a literature estimate of abundance, we averaged the cohesin regulator numbers quantified in HeLa cells by mass spectrometry^37^ and fluorescence correlation spectroscopy (FCS).^36^ Absolute protein copy numbers were converted to genomic densities by considering a ∼19.539*Gb* average genome size, as previously reported for diploid HeLa cells.^82^ Since FCS measurements of PDS5 abundance are to our knowledge currently lacking, we used as an alternative estimate the mean stoichiometric PDSA/B-to-WAPL ratio reported in HeLa immunoprecipitation assays via SMC1 and SMC3 pulldowns.^38^ We similarly curated Fluorescence Recovery After Photo bleaching (FRAP) data to obtain bound fractions and residence times for cohesin regulators in HeLa cells from the following publications: RAD21 from Holzmann et al.^36^; NIPBL from Rhodes et al.,^28^; WAPL and PDS5 from Ladurner et al*.*^22^ These values (Table 1) were employed to ascertain the reaction network of unperturbed HeLa cells.

#### Cohesin biochemical network assumptions

1. RAD21 may be taken as a proxy for the core cohesin complex:
  • Based on structural insights that RAD21 acts as a “nexus” for the recruitment of cohesin regulatory factors NIPBL, PDS5, and WAPL.^13^
  • The dynamic residence time of RAD21, SMC3, SMC1, and SA1 are all similarly in the tens of minutes range.^29^^,^^35^^,^^64^
2. Cohesin regulators do not bind chromsatin in the absence of RAD21:
  • NIPBL^28^: reports that RAD21 depletion releases the majority of (but not all) NIPBL from chromatin, suggesting that RAD21 association is the dominant pathway for loading NIPBL onto chromosomes. This is also consistent with the disappearance of NIPBL-associated ChIP-seq peaks upon removal of cohesin.^73^
  • WAPL^35^: reports that WAPL cannot be detected on chromatin in RAD21-depleted cells.
  • PDS5^83^: reports that the recruitment of PDS5 of chromatin is drastically reduced in cells not expressing RAD21. This is also consistent with reports that disrupting the RAD21-PDS5 binding interface largely abolishes the association of PDS5 with chromatin.^13^
3. Co-bound states involving the simultaneous association of multiple regulators are neglected:
  • PDS5+NIPBL: Reports that PDS5 competes with NIPBL for RAD21 binding^25^^,^^26^ support this hypothesis.
  • WAPL+NIPBL: Supported by,^84^ which report a small (but non-zero) co-bound population in WAPL-NIPBL co-IP experiments.
  • WAPL+PDS5: Although WAPL, RAD21 and PDS5 have been recently suggested to be able to form a tripartite complex using AlphaFold,^24^ experimental observations in reconstituted protein assays have revealed that WAPL may also stably associate with RAD21 in the absence of PDS5.^34^ Similarly, the chromatin association of PDS5 *in vivo* was largely unaffected by WAPL depletion^85^ arguing against a substantial population of WAPL and PDS5 being concomitantly bound to cohesin, despite a reported role for PDS5 in the recruitment of WAPL^86^*. In vitro* studies have further reported conflicting evidence of direct interactions between WAPL and PDS5,^13^^,^^23^ although co-IP experiments support the existence of WAPL-PDS5A co-binding in presence of RAD21.^34^^,^^35^ In light of this uncertainty, we neglect the simultaneous binding of PDS5 and WAPL onto RAD21 as a first approximation. However, we also explore a model with a strictly co-bound WAPL+PDS5 state and describe an experimental signature to unambiguously assess its relevance.
4. The A and B paralogs of PDS5 may be amalgamated:
  • First, the two paralogs associate with cohesin in a mutually-exclusive fashion.^87^ Second, studies have suggested that PDS5A/B share a large structural and functional redundancy.^88^ Future experiments would be required to determine any differential effects on extrusion^84^^,^^89^ as distinct FRAP measurements for PDS5A and PDS5B are, to our knowledge, currently lacking.
5. We consider models with 8 non-zero transition rates and four loaded states:
  • This precludes the study of potentially interesting models with additional transitions, e.g., transient loading of non-extrusive cohesin is neglected,^22^ along with potential WAPL-independent cohesin dissociation.^68^

#### Rate mapping

Cohesin state transition rates were inferred from the experimentally-measured bound fractions and residence times of individual proteins. Assuming mass action kinetics, we derived a system of coupled ordinary differential equations (ODEs) for the populations of RAD21, NIPBL, PDS5 and WAPL. This system of ODEs involves 8 unknown cohesin state transition rates (Figure 1). Accordingly, we derived 8 mathematical constraints from the chromatin residence time and bound fraction for each molecular species. Combining these, we arrive at a system of 8 linear equations, which may be solved symbolically to obtain explicit expressions for the transition rates as rational functions of the protein absolute abundances, residence times, and bound fractions (Table 1; Methods S1, Rate mapping procedure).

#### Lattice model for extrusion kinetics

We simulated loop extrusion as a discrete-time Markov process on a 1D lattice with timestep *τ*_1*D*_ at a genomic resolution of *l*∼2.5*kb* per site using the discrete-time-extrusion package (https://github.com/Fudenberg-Research-Group/discrete-time-extrusion,^75^). When extruders are loaded, a left and right leg are placed on adjacent lattice sites. During each update step, a given extruder may be stochastically loaded, unloaded, or transition into a new loaded state within the cohesin biochemical network (*RN*, *R*, *RP*, *RW*) based on a simple discrete-time kinetic Monte-Carlo sampling of the five-state reaction network. After the possible state update, if an extruder is in the active NIPBL-bound (*RN*) state, each leg of the extruder moves one lattice site outwards, provided that adjacent lattice sites are unoccupied. After each update, the positions of all extruder legs and extruder states are recorded. For simplicity, we assumed that the corresponding transition rates between different cohesin states are uniform across all sites—i.e., that cohesin physico-chemical properties are independent of the local genomic context—and may thus be identically set to the respective values inferred from the rate mapping procedure (Table S1).

Denoting by [*RN*] the total numbers of RAD21 molecules bound by NIPBL at equilibrium, the mean extrusion rate at steady state reads as *v* = 2*L*/*τ*_1*D*_×[*RN*]/[*R*]_*loaded*_, where [*R*]_*loaded*_ = [*RN*]+[*R*]+[*RP*]+[*RW*] indicates the total equilibrium population of chromatin-associated RAD21 and the factor 2 accounts for the two legs of the cohesin complex. The bursty extrusion model predicts the value of the active-to-loaded extruder ratio as [*RN*]/[*R*]_*loaded*_≃26% in wild-type HeLa cells. Using the typical extrusion rate of *v*∼1*kb*/*s* estimated for cohesin by single-molecule imaging *in vitro*, we may thus infer that each lattice step *τ*_1*D*_ corresponds to approximately ∼1.25*s* of physical time.

Using these values, the model predicts a transition frequency into the NIPBL-bound state of about 0.3 times per minute (Figure 2A). This rate is slightly slower than an experimental value of ∼1/min, as inferred from the ∼0.5/min rate of cohesin direction changes reported in single-molecule *in vitro* assays,^40^ where the factor of 2 accounts for the two possible extrusion directions after each NIPBL binding event. Note that since extrusion traces lacking a direction switch were excluded from analysis in,^40^ their estimated experimental rate of ∼1/min likely provides an upper bound for the average transition frequency into the NIPBL-associated state.

#### Polymer model of multi-state extrusion

We model a 500 Mb-long chromatin region as a linear polymer comprising 200,000 monomers of 2.5*kb* each, such that each site from the lattice model corresponds to a unique individual bead in the chromatin chain. As in previous investigations,^43^ we use a spatial extent of each bead of *σ*∼50*nm*, consistent with recent estimates of chromatin compaction of 50*kb*/*μm* in eukaryotes.^90^ To model the impact of extruders on 3D polymer conformations, we generated extruder positions using the 1D lattice model and created additional bonds between pairs of monomers occupied by the two legs of each extruder. Polymer simulations were run using the polychrom-hoomd package (https://github.com/open2c/polychrom-hoomd,^76^), based on the HooMD molecular dynamics engine,^91^ considering a 20% polymer volume fraction combined with a polynomial soft excluded-volume potential^92^ (see https://github.com/fudenberg-research-group/multistate-extrusion-networks and https://github.com/open2c/polychrom-hoomd for full implementation details). This concentration amounts to an approximate chromatin density of 0.01*bp*/*nm*^3^, consistent with the typical orders of magnitude reported in HeLa cells.^93^ Numerical integration was performed using dissipative particle dynamics (DPD) with a dimensionless 3D integration timestep *τ*_3*D*_ = 0.005.^94^ At long times (⪰100*s*), the computed (Rouse) diffusion coefficient of individual monomers reads as $D_{Rouse}^{sim}∼0.3\;\sigma^{2}/\tau_{3D}^{0.5}$ (Figure S5A). The mapping of simulation to experimental times is performed by matching $D_{Rouse}^{sim}$ to the experimental value $D_{Rouse}^{\exp}=0.01\;\mu m^{2}/s^{0.5}$ estimated in yeast chromatin,^95^ which yields *τ*_3*D*_∼5*ms*. Alternatively, the presence of cohesin with wild-type extrusion parameters (Table 1) leads to a simulated super-Rousean anomalous diffusion coefficient $D_{extrusion}^{sim}∼0.1\;\sigma^{2}/\tau_{3D}^{0.675}$ at short times (⪯100*s*), which may be compared to the experimental value $D_{extrusion}^{\exp}\approx 0.0075\;\mu m^{2}/s^{0.675}$ estimated in CTCF-depleted mESCs over a similar time interval^96^ (Figure S5A). This procedure similarly leads to *τ*_3*D*_∼5*ms*, which evidences the relative robustness of this time mapping. The number of MD steps performed between each extruder update is then given by *N*_3*D*/1*D*_ = *τ*_1*D*_/*τ*_3*D*_ = 250. To assess the sensitivity of our predictions to this number, we analyzed the mean-squared error in the simulated contact frequency versus distance curve (*P*(*s*); see below), compared to the experimental Hi-C profile observed in CTCF-depleted HeLa cells,^18^^,^^19^^,^^21^ across 10 different *N*_3*D*/1*D*_ values in the range [100, 1000] (Figures S5B and S5C). This procedure yields an optimal agreement between theory and experiment for *N*_3*D*/1*D*_ = 300, corresponding to a mean extrusion rate of *v* ∼850*bp*/*s* – highly consistent with biophysically-inferred parameters (*N*_3*D*/1*D*_ = 250, *v*∼1*kb*/*s*).

### Quantification and statistical analysis

#### Contact frequency versus distance curves

For the calculation of contact frequency versus distance (*P*(*s*)) curves, we used the monomerResolutionContactMapSubchains function from the contact_maps module as implemented in the polykit package (https://github.com/open2c/polykit,^77^). Contact maps were computed at the single monomer level using a default cutoff distance *R*_*c*_ = 2.3*σ*∼115*nm*, consistent with the typical capture radius assumed in standard Hi-C experiments.^97^ *P*(*s*) scaling curves were then directly computed from the maps using the expected_cis function of the cooltools library.^79^ To obtain the experimental contact frequency distance curves, *P*_*exp*_(*s*), we re-processed experimental Hi-C datasets from HeLa cells^18^^,^^19^^,^^21^ using the distiller pipeline (https://github.com/open2c/distiller-nf,^81^), extracting contacts with pairtools (https://github.com/open2c/pairtools,^80^), and binning to 1*kb* resolution with cooler (https://github.com/open2c/cooler,^78^). To quantify agreement between simulations and experiment, a goodness-of-fit parameter (*R*^2^) was then defined as

$$R^{2}=1-\frac{\sum_{s}{\left[P_{\exp}\left(s\right)-P\left(s\right)\right]}^{2}}{\sum_{s}{\left[P_{\exp}\left(s\right)-{\bar{P}}_{\exp}\right]}^{2}}$$

where the summation and average (${\bar{P}}_{\exp}$) are both performed over the genomic range [50*kb*, 10*Mb*] using 9,500bins. The lower bound of 50*kb* was chosen to lie above the threshold of 40*kb*, beyond which HindIII-based Hi-C data appears unaffected by restriction and ligation artifacts resulting from the experimental library preparation protocol.^98^ Accordingly, simulated *P*(*s*) were normalized to align with the corresponding experimental curve *P*_*exp*_(*s*) at *s* = 50*kb*.

#### Numerical microscopy & vermicelli score

To matching the typical scanning resolution of confocal microscopes, 3D voxels of dimension 100*nm* × 100*nm* × 100*nm* are used for the binning of cohesin and DNA spatial positions. The resulting cubic rasters feature 50 voxels along each axis, corresponding to a total field of view with linear dimension 5*μm*. To mimic the presence of unloaded extruders, additional diffusive cohesins are randomly and uniformly distributed throughout the sample, with numbers matching the unloaded population of RAD21 predicted by the bursty extrusion model in each condition. A Gaussian convolution filter with standard deviation of 250*nm* is subsequently applied to approximate the Airy point spread function. The Pearson correlation score between the diffracted cohesin and DNA signal is finally computed by averaging across 5000 MD frames obtained from 5 independent simulations for each sample.

## Supplemental information

Document S1. Figures S1–S11 and Tables S1 and S3

Methods S1. Technical details of the derivation of chemical-transition-rate mapping equations and proof of unicity of network equilibrium state, related to STAR Methods

Document S2. Article plus supplemental information
