# Supplementary material for: The physical chemistry of interphase loop extrusion
Source: Cell Genom. 2025 Dec 10;6(3):101098. doi: 10.1016/j.xgen.2025.101098 (PMC12985376; doi:10.1016/j.xgen.2025.101098)
Supplement: Document S1. Figures S1–S11 and Tables S1 and S3 [file mmc1.pdf]

Cell Genomics, Volume *6*

Supplemental information

The physical chemistry of interphase loop extrusion

Maxime M.C. Tortora and Geoffrey Fudenberg

State transition rates	
k_{on}	$3.4 \times 10^{-8}\,\mathrm{s}^{-1}\,\mathrm{molecule}^{-1}$
k_{off}	$9.2 \times 10^{-3}\,\mathrm{s}^{-1}$
k_{NR}	$1.4 \times 10^{-2}\,\mathrm{s}^{-1}$
k_{RN}	$2.0 \times 10^{-7}\,\mathrm{s}^{-1}\,\mathrm{molecule}^{-1}$
k_{RP}	$2.7 \times 10^{-7}\,\mathrm{s}^{-1}\,\mathrm{molecule}^{-1}$
k_{PR}	$7.4 \times 10^{-3}\,\mathrm{s}^{-1}$
k_{PW}	$1.6 \times 10^{-7}\,\mathrm{s}^{-1}\,\mathrm{molecule}^{-1}$
k_{WP}	$1.4 \times 10^{-7}\,\mathrm{s}^{-1}\,\mathrm{molecule}^{-1}$

TABLE S1. **Equilibrium rates of the bursty extrusion model for wild-type HeLa cells (related to Fig. 1).** Numerical values are computed from the analytical expressions Eqs. (24)–(31), substituting for the experimentally-measured biochemical parameters summarized in Table 1 of the main text.

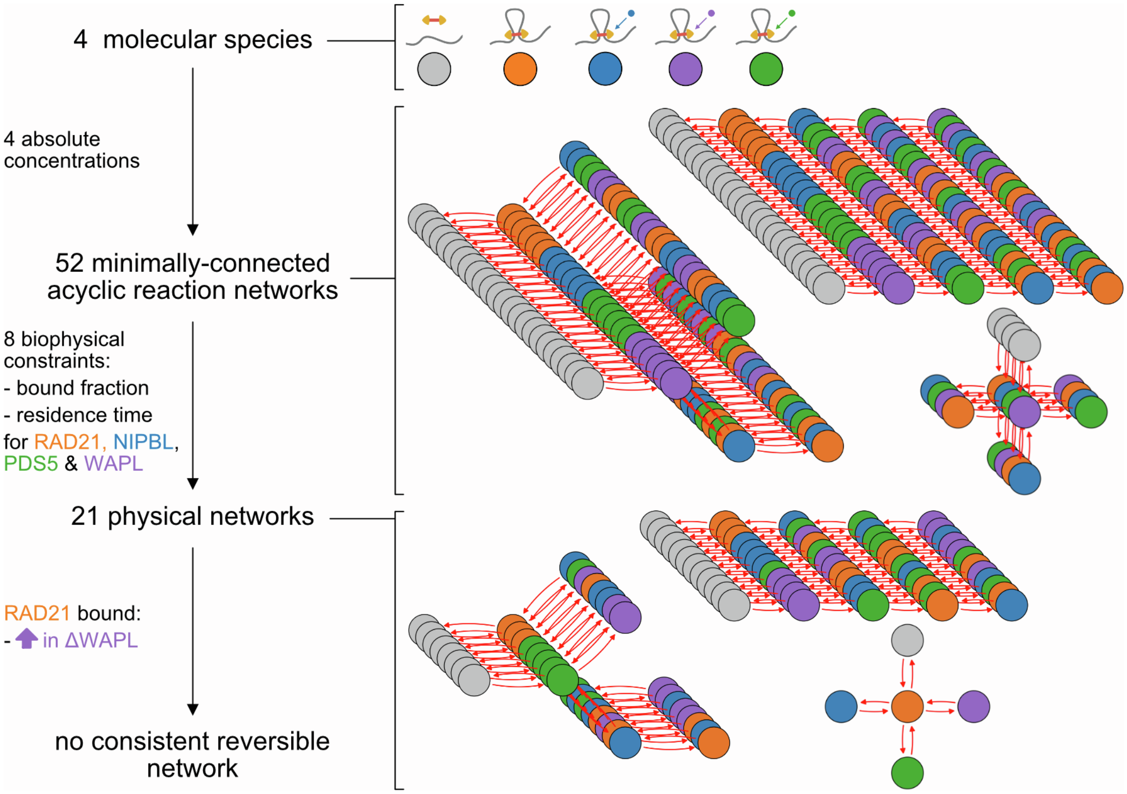

FIG. S1. **Chromatin entry and exit involves distinct cohesin molecular pathways (related to Fig. 1).**

To systematically explore and rule out the possibility of cohesin loading and unloading via a single pathway, we apply the same decimation procedure as used in Fig. 1b of the main text to all possible acyclic, fully reversible networks with minimal number of edges (8). While 21 networks can be found with non-negative rates, none of these networks lead to an increase in the loaded fraction of RAD21 upon depletion of WAPL — or, more generally, of any of the other cohesin regulators. Thus, no fully reversible networks are consistent with experimental observations

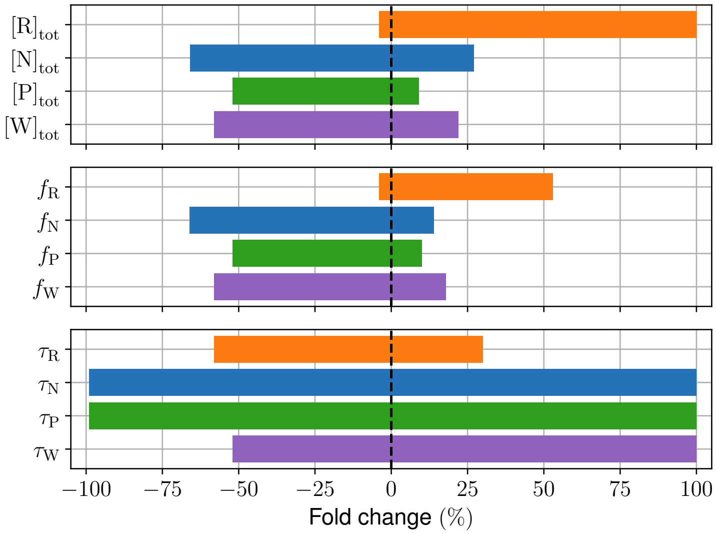

FIG. S2. **Bursty extrusion model is robust to uncertainties in experimental measurements (related to Fig. 1).**

To investigate the stability of the bursty extrusion model to changes in cohesin parameters *in vivo*, we systematically varied the nuclear abundance, bound fraction and chromatin residence time of RAD21 and the regulators NIPBL, PDS5 and WAPL, and reported the regimes in which the pruning procedure in Fig. 1b resulted in its identification as the unique viable candidate network (colored regions). The large tolerance intervals obtained for the majority of parameters evidences the robustness of the model, with the most stringent constraint stemming from the fact that the total amount of chromatin-bound regulators may not exceed the overall population of loaded cohesins, i.e., $f_N\,[\mathrm{N}]_{\mathrm{tot}} + f_P\,[\mathrm{P}]_{\mathrm{tot}} + f_W\,[\mathrm{W}]_{\mathrm{tot}} < f_R\,[\mathrm{R}]_{\mathrm{tot}}$. This requirement results from our assumption of strict exchange kinetics between cohesin regulators, and could be potentially relaxed by accounting for the additional possibility of co-bound states (c.f. Fig. S8).

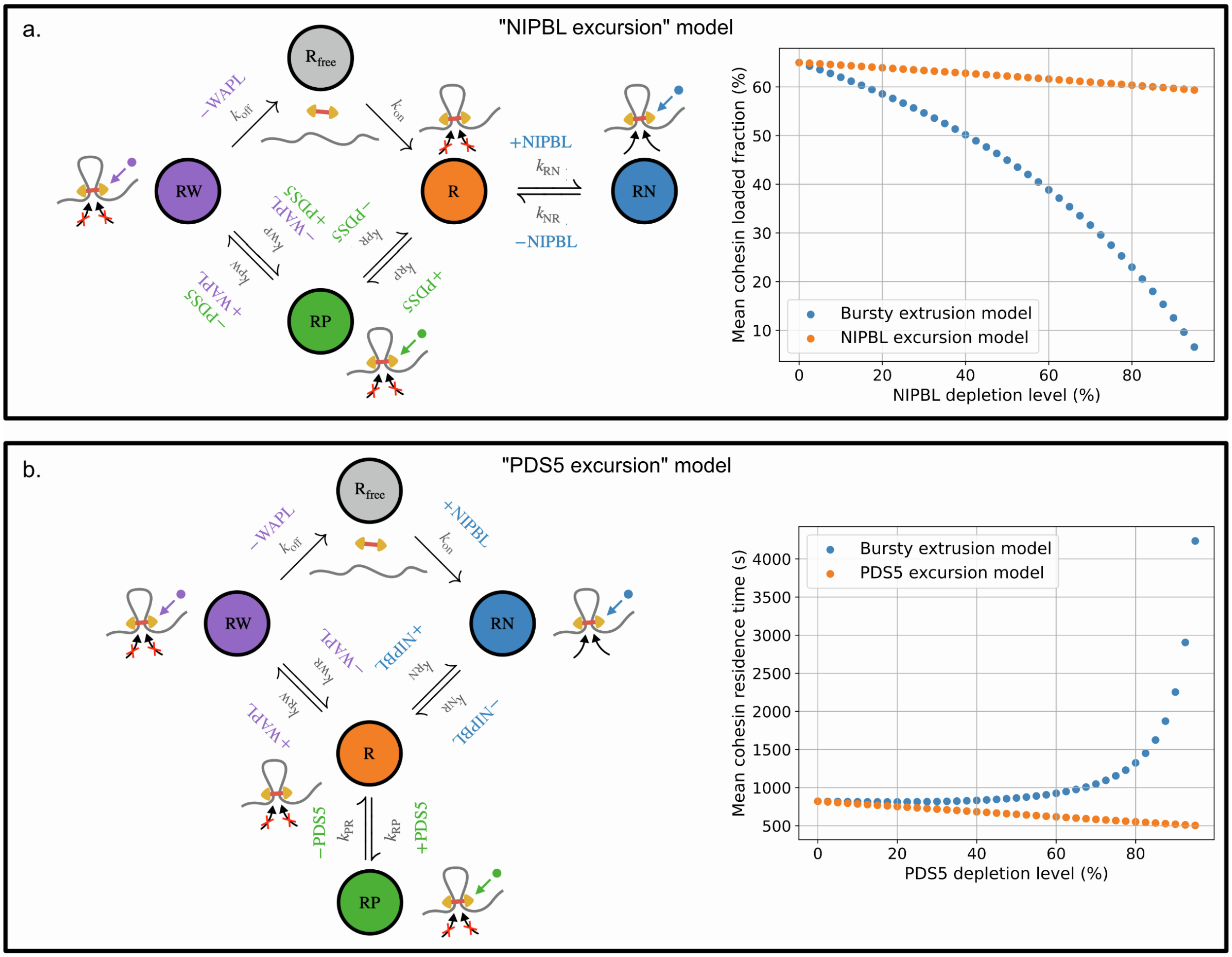

FIG. S3. **Alternate topologies are inconsistent with experiments (related to Fig. 1).** **a.** NIPBL excursion model, where NIPBL is not involved with loading but instead binds reversibly after the core complex has loaded onto chromatin. For this topology, NIPBL depletion does not substantially lower the loaded fraction, unlike experimental observations. The inconsistency of this alternate topology provides mathematical support for the role of NIPBL in productive cohesin loading in an extrusion cycle. **b.** PDS5 excursion model, where PDS5 reversibly binds the core complex and does not promote WAPL binding. For this topology, PDS5 depletion actually slightly lowers RAD21 residence time, instead of increasing RAD21 residence time as observed experimentally. The inconsistency of this alternate topology argues that PDS5 is positioned along the reaction cycle in such a way to influence unloading rates.

			Mutant depletion		
			ΔNIPBL	ΔPDS5	ΔWAPL
**Cohesin accessory protein**	RAD21	Bound fraction	*In vivo* [18]	*In vivo* [18]	*In vivo* [18]
		Residence time	*In vitro* [40]	*In vivo* [18]	*In vivo* [18]
	NIPBL	Bound fraction			*In vivo* [28]
		Residence time	*In vitro* [40]		*In vivo* [28]
	PDS5	Bound fraction			
		Residence time			
	WAPL	Bound fraction			
		Residence time			

TABLE S2. **Predicted impacts of accessory protein depletions on their chromatin association dynamics in the bursty extrusion model (related to Fig. 4).**
Recapitulative table of the role of various cohesin accessory protein depletions (columns) on the chromatin-associated fraction and residence time of other proteins (rows). Red (resp. blue) colors signify that the model predicts an increase (resp. decrease) of the corresponding quantity in the different mutants relative to its magnitude in wild-type HeLa cells. Gray shades mark a lack of significant deviation from the wild-type value. References point to experimental studies reporting *in vivo* or *in vitro* validation of the predicted changes, wherever available. We note that NIPBL depletion leads to a drastic reduction in the bound fractions of PDS5 and WAPL, consistent with a significant inhibition of cohesin loading, but is associated with a more moderate drop (10-20%) in their respective cohesin residence times (Fig. 4c of the main text).

			**Mutant depletion**		
			ΔNIPBL	ΔPDS5	ΔWAPL
**Cohesin accessory protein**	RAD21	Bound fraction	*In vivo* [18]	*In vivo* [18]	*In vivo* [18]
		Residence time	*In vitro* [40]	*In vivo* [18]	*In vivo* [18]
	NIPBL	Bound fraction			*In vivo* [28]
		Residence time	*In vitro* [40]		*In vivo* [28]
	PDS5	Bound fraction			
		Residence time			
	WAPL	Bound fraction			
		Residence time			

TABLE S3. **Same as Table S2 for the PDS5-WAPL co-bound model (Fig. S8a, related to Fig. 4).**

Note that the main qualitative difference with the bursty extrusion model lies in the residence time of PDS5 in WAPL-depleted cells, which decreases relative to wild-type in the PDS5-WAPL co-bound model, but increases in the case of the strict subunit exchange assumed by the bursty extrusion model (Fig. S8d).

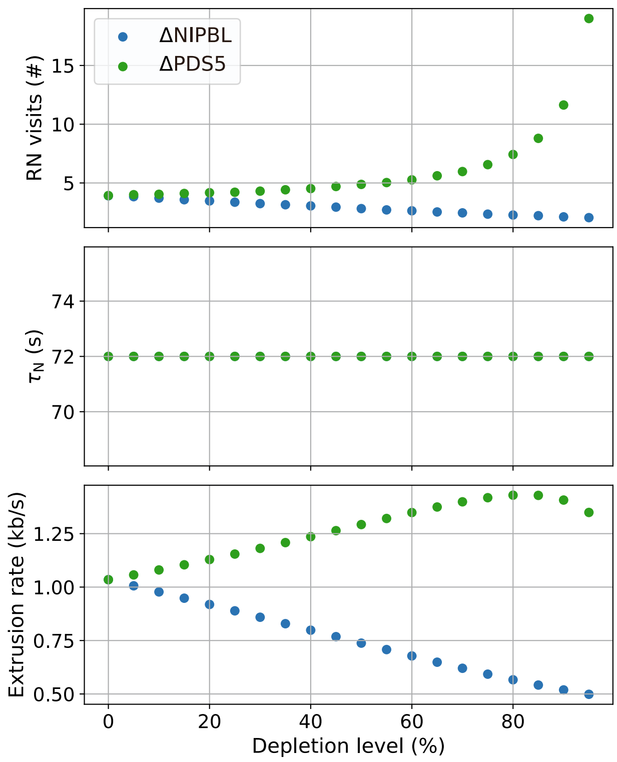

FIG. S4. **Frequency of visits to the RN state governs extrusion rate (related to Fig. 2). (Top)** Average number of transitions into the actively-extruding, NIPBL-bound (RN) state per cohesin loading window, as a function of PDS5 and NIPBL depletion levels. **(Center)** Although the cohesin residence time of NIPBL (τ_N) is insensitive to the abundance of regulators (c.f. Table S2), the mean time fraction that loaded cohesins spend associated with NIPBL is controlled by the frequency of visits to RN. This translates into a higher average extrusion rates after PDS5 depletion and lower rates after NIPBL depletion **(Bottom)**.

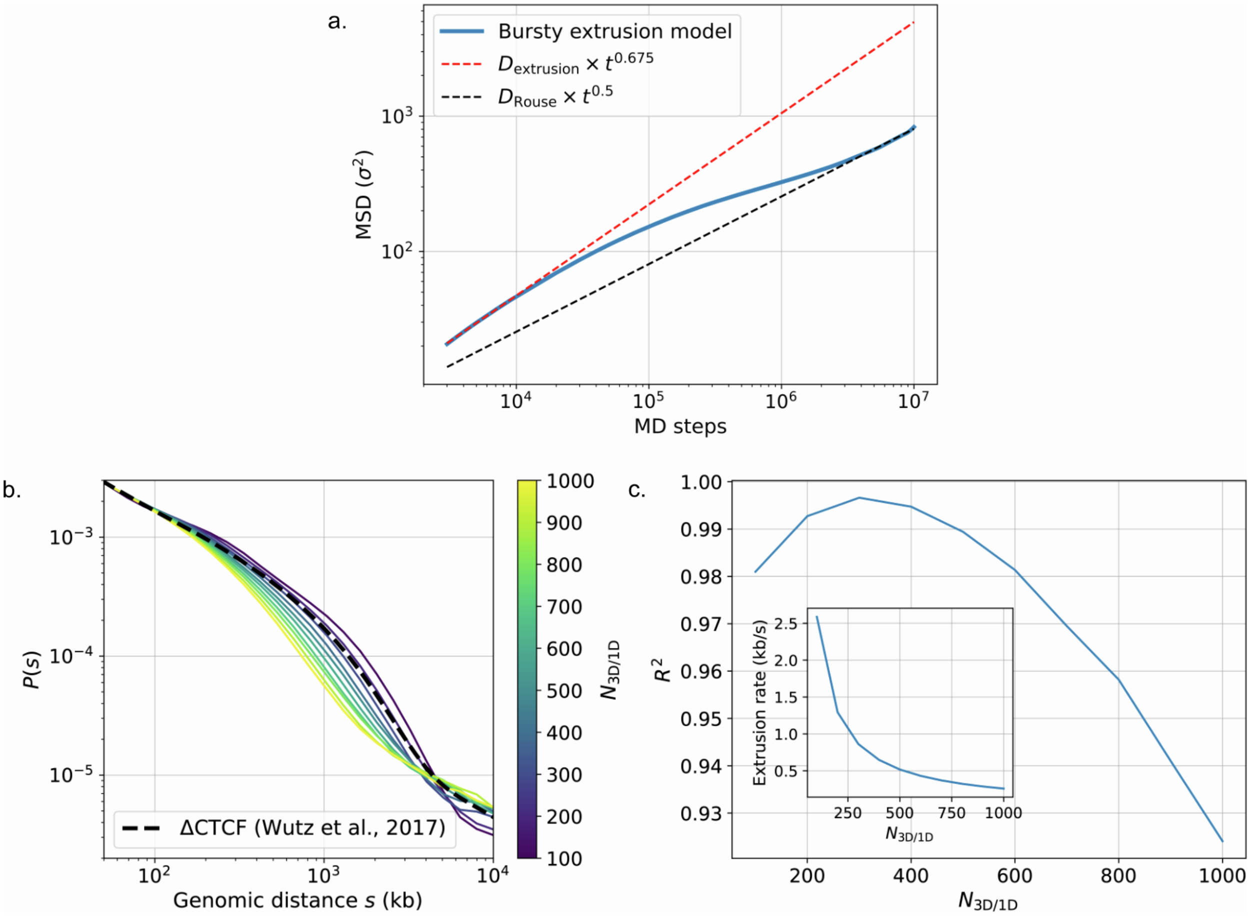

FIG. S5. **Time mapping & influence of loop extrusion rate (related to Fig. 3).**
a. Mean-squared displacement (MSD) of individual monomers as predicted by the bursty extrusion model at wild-type HeLa protein expression levels ($\sigma = 50\,\mathrm{nm}$). The correspondence between model and experimental time units (1 MD step $\sim 5\,\mathrm{ms}$) is obtained by comparing the slopes of the long- and short-time asymptotes to the respective experimental values $D_{\mathrm{Rouse}} \simeq 0.01\,\mu\mathrm{m}^2/\mathrm{s}^{0.5}$ and $D_{\mathrm{extrusion}} \simeq 0.0075\,\mu\mathrm{m}^2/\mathrm{s}^{0.675}$, as estimated in budding yeast [95] and CTCF-depleted mESCs [96]. **b.** Computational contact-vs-distance curves ($P(s)$) at different ratios of 3D-to-1D steps ($N_{\mathrm{3D/1D}}$). Dashed line: experimental profile obtained in ΔCTCF mutants [18]. **c.** Mean-squared R coefficient in the model vs. experimental P(s) curves, averaged over the distance range [50 kb: 10,000 kb]. Inset: Correspondence between $N_{\mathrm{3D/1D}}$ and mean extrusion rate (v) in wild-type HeLa cells.

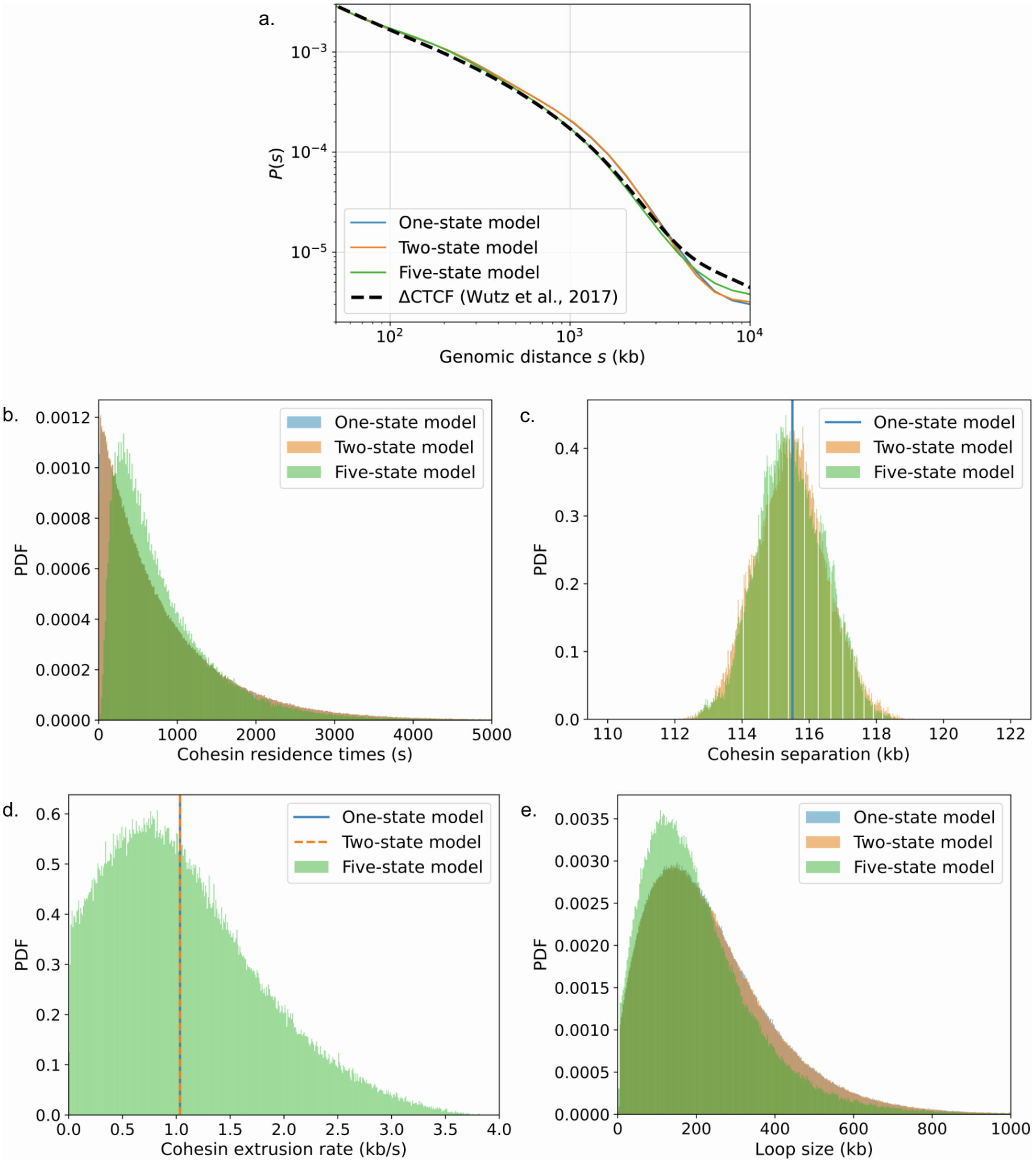

a.
P(s)
Genomic distance s (kb)
One-state model
Two-state model
Five-state model
ΔCTCF (Wutz et al., 2017)
b.
PDF
Cohesin residence times (s)
c.
Cohesin separation (kb)
d.
Cohesin extrusion rate (kb/s)
e.
Loop size (kb)

FIG. S6. **Bursty extrusion (a.k.a. five-state) model provides additional sources of heterogeneity absent from previous cohesin models (related to Fig. 3).**
One state and two-state models are parameterized to have the same average extrusion rate, cohesin residence time and number of loaded extruders as the bursty extrusion model, but respectively assume a constant translocation rate with or without immediate cohesin reloading. **a.** Contact frequency versus distance curves of the different models. Quantitative agreement of the bursty extrusion model with experimental ΔCTCF data [18] is noticeably closer than its one- and two-state counterparts ($R^2 > 0.99$ vs. $R^2 = 0.98$), with the predictions of the latter two being largely undistinguishable. **b.** The distribution of residence times of the bursty extrusion model deviates from the simple exponential profile of the one- and two-state models. **c.** The distribution of the average separation (i.e., inverse loaded density) of extruders displays a similar level of heterogeneity in the two- and five-state models, which is lacking in the one-state model with immediate reloading. **d.** The distribution of cohesin extrusion rates, averaged over the chromatin residence time of each extruder, evidences an additional source of heterogeneity not present in one- or two-state models. **e.** Distributions of loop sizes are nonetheless qualitatively similar across the three models.

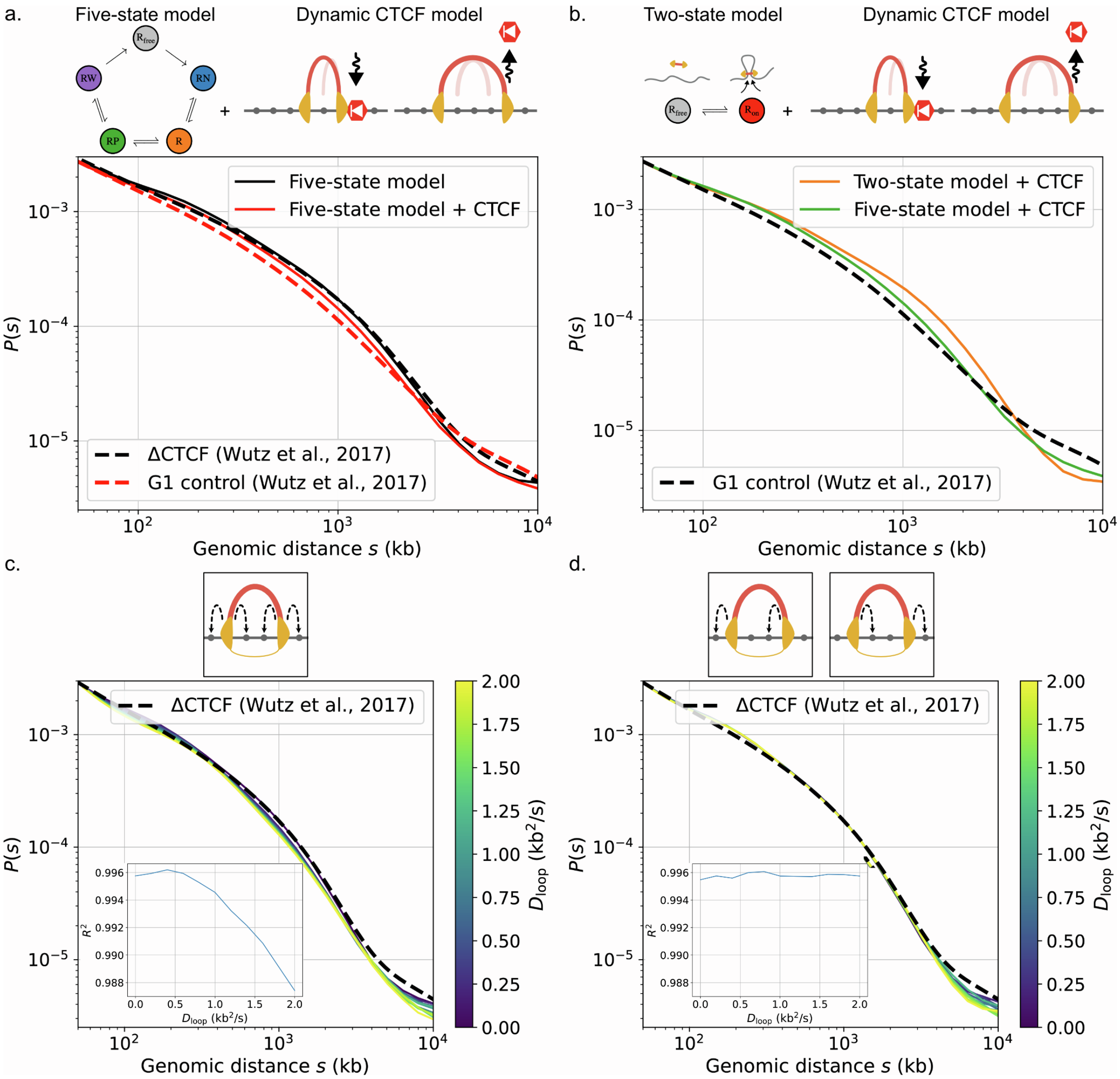

a.
Five-state model
Dynamic CTCF model
Five-state model
Five-state model + CTCF
ΔCTCF (Wutz et al., 2017)
G1 control (Wutz et al., 2017)
P(s)
Genomic distance s (kb)
b.
Two-state model
Dynamic CTCF model
Two-state model + CTCF
Five-state model + CTCF
G1 control (Wutz et al., 2017)
c.
ΔCTCF (Wutz et al., 2017)
D_loop (kb²/s)
d.
ΔCTCF (Wutz et al., 2017)

FIG. S7. **Roles of extrusion kinetics, CTCF and loop diffusion on contact frequency versus distance (related to Fig. 3).**

a-b. Consequences of dynamic CTCF barriers with experimentally-calibrated parameters ($[\mathrm{CTCF}]_{\mathrm{tot}} = 130,000$ [36,37], $f_{\mathrm{CTCF}} = 0.7$ [50], $\tau_{\mathrm{CTCF}} = 120\,\mathrm{s}$ [50]). Uni-directional barriers were positioned randomly across the genomic region of interest, and simulated as in [48]. **a.** Coupling the bursty extrusion (a.k.a. five-state) model with dynamic CTCF barriers predicts a mild reduction of genomic contacts in the range [100 kb: 4,000 kb], improving quantitative agreement with experiments in control G1 HeLa cells ($R^2 = 0.98$). **b.** In contrast, coupling CTCF to previous two-state models — in which loaded cohesins continuously extrude DNA with the same average translocation rate $v = 1\,\mathrm{kb\,s^{-1}}$ as in the bursty extrusion model — underestimated the reduction in P(s) and resulted in a noticeably worse fit to experimental data ($R^2 = 0.94$). **c-d.** Consequences of two varieties of diffusion. **c.** Assuming that each leg of the complex may stochastically and independently diffuse regardless of cohesin state, we find that optimal agreement with experimental data is reached at loop diffusion rates $D_{\mathrm{loop}} \sim 0.4\,\mathrm{kb^2/s}$ in CTCF-depleted HeLa cells. **d.** Conversely, assuming that both legs strictly diffuse in tandem does not yield any measurable effects on simulated $P(s)$ curves.

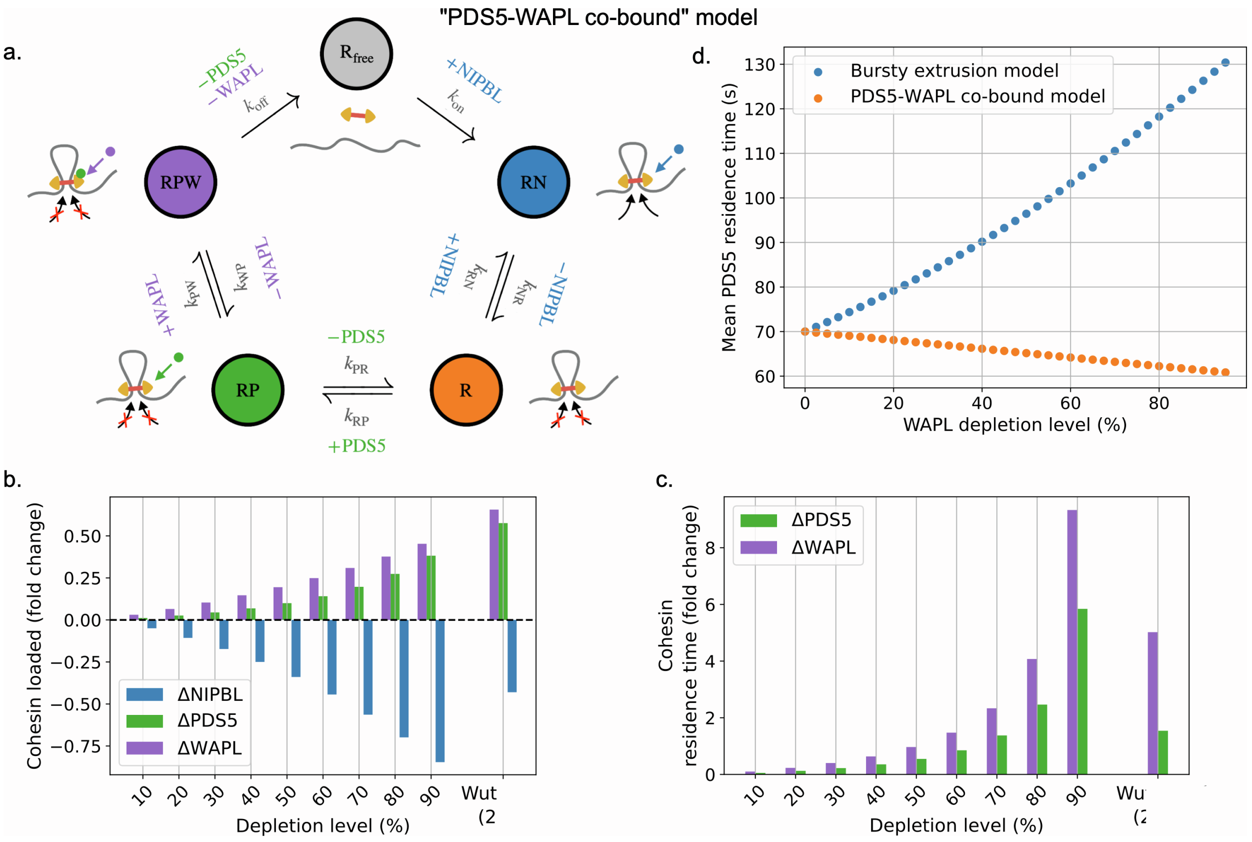

FIG. S8. **A strict co-binding model for cohesin unloading by PDS5 and WAPL (related to Fig. 4).**

a. Chemical reaction cycle for the PDS5-WAPL co-bound model. While the reaction cycle is largely similar to that of the bursty extrusion model, it differs from the strict regulator exchange considered in the main text by assuming that PDS5 and WAPL can simultaneously bind RAD21, and are jointly required for the unloading of the core complex. **b.** Relative change in cohesin loaded fraction with regulator depletion in simulations and experiments (c.f. Figs. 4e–f of the main text). The simulated effects of PDS5 depletion are now more similar to those of WAPL, while NIPBL depletion has a similar impact to the strict exchange model. **c.** Relative change in cohesin residence time with WAPL or PDS5 depletion. The strictly co-bound model significantly overshoots experimentally-observed increases in cohesin residence time after PDS5 or WAPL RNAi. **d.** Simulated PDS5 residence time as a function of WAPL depletion levels, which suggests the experimental characterization of PDS5 residence time after WAPL depletion (via, e.g., FRAP or single-particle tracking) as a useful metric to assess the validity of the co-bound versus strict exchange models.

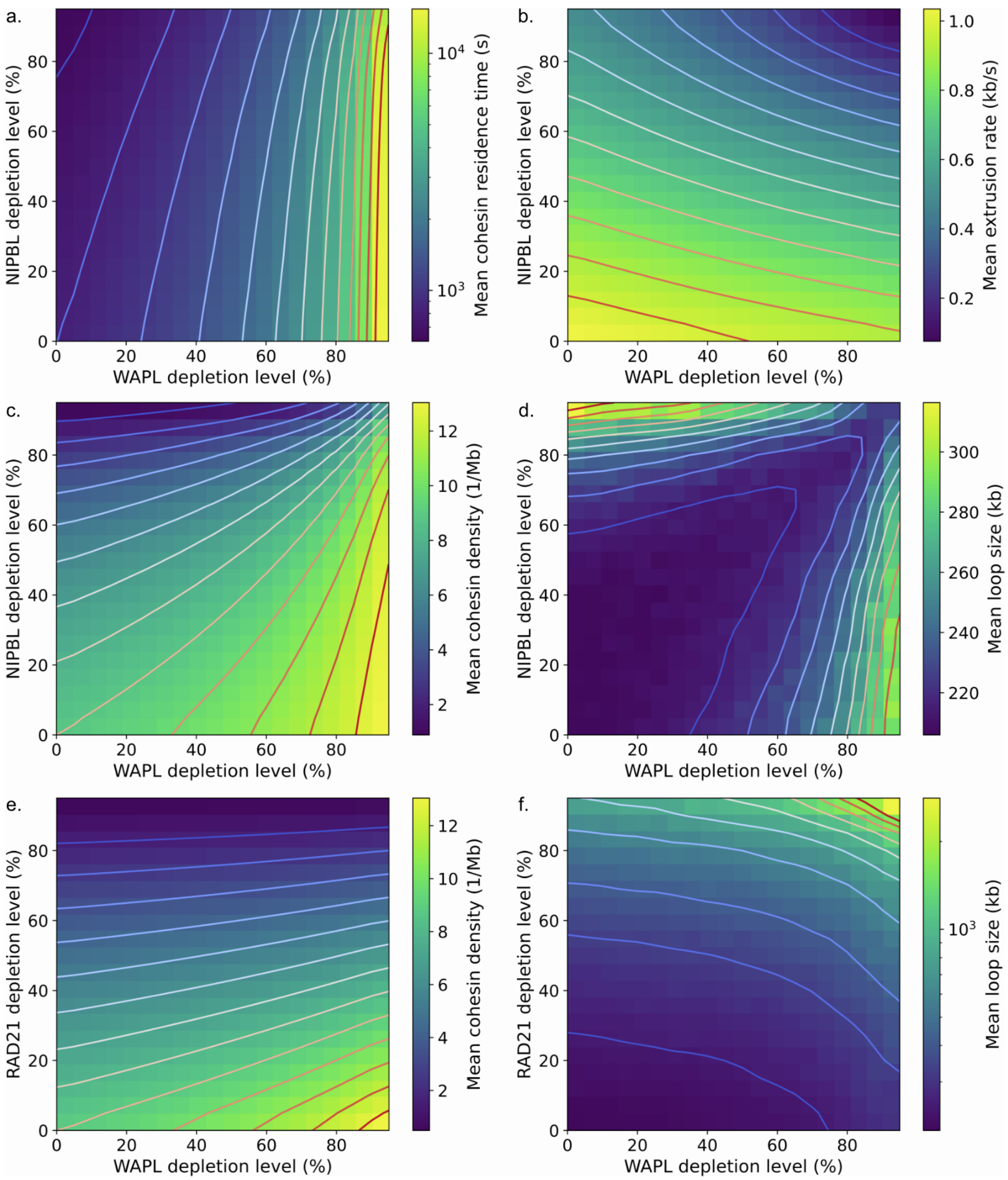

a.
NIPBL depletion level (%)
WAPL depletion level (%)
Mean cohesin residence time (s)
10^4
10^3
0
20
40
60
80
b.
NIPBL depletion level (%)
WAPL depletion level (%)
Mean extrusion rate (kb/s)
1.0
0.8
0.6
0.4
0.2
c.
NIPBL depletion level (%)
WAPL depletion level (%)
Mean cohesin density (1/Mb)
12
10
8
6
4
2
d.
NIPBL depletion level (%)
WAPL depletion level (%)
Mean loop size (kb)
300
280
260
240
220
e.
RAD21 depletion level (%)
WAPL depletion level (%)
Mean cohesin density (1/Mb)
f.
RAD21 depletion level (%)
WAPL depletion level (%)
Mean loop size (kb)
10^3

FIG. S9. **Bursty extrusion model evidences a compensatory role for NIPBL and WAPL (related to Fig. 4).**

a–d. Heatmaps indicating changes in various metrics of extrusion activity as a function of NIPBL and WAPL co-depletion levels. Lines show iso-levels of the indicated quantity. NIPBL does not balance the impact of WAPL on residence time, as it only marginally impacts residence time (**a**). However, NIPBL depletion generally leads to a considerable reduction in the mean translocation rate (**b**), and can balance the effects of WAPL depletion for both numbers of cohesin per megabase (i.e. cohesin loaded density, **c**). Thus, NIPBL and WAPL co-depletion generally leads to higher cohesin residence times, but lower extrusion rates — and is thus able to rescue loop sizes when both complexes are down-regulated in similar proportions (**d**). **e–f.** Same as (c) and (d) for RAD21 and WAPL co-depletion. Unlike NIPBL, RAD21 depletion generally has a limited impact on both residence time and translocation rate (Fig. 4d of the main text), and cannot compensate the effects of WAPL. Thus, while cohesin loaded density can be potentially balanced by simultaneous down-regulation of RAD21 and WAPL (**e**), loop sizes generally cannot (**f**).

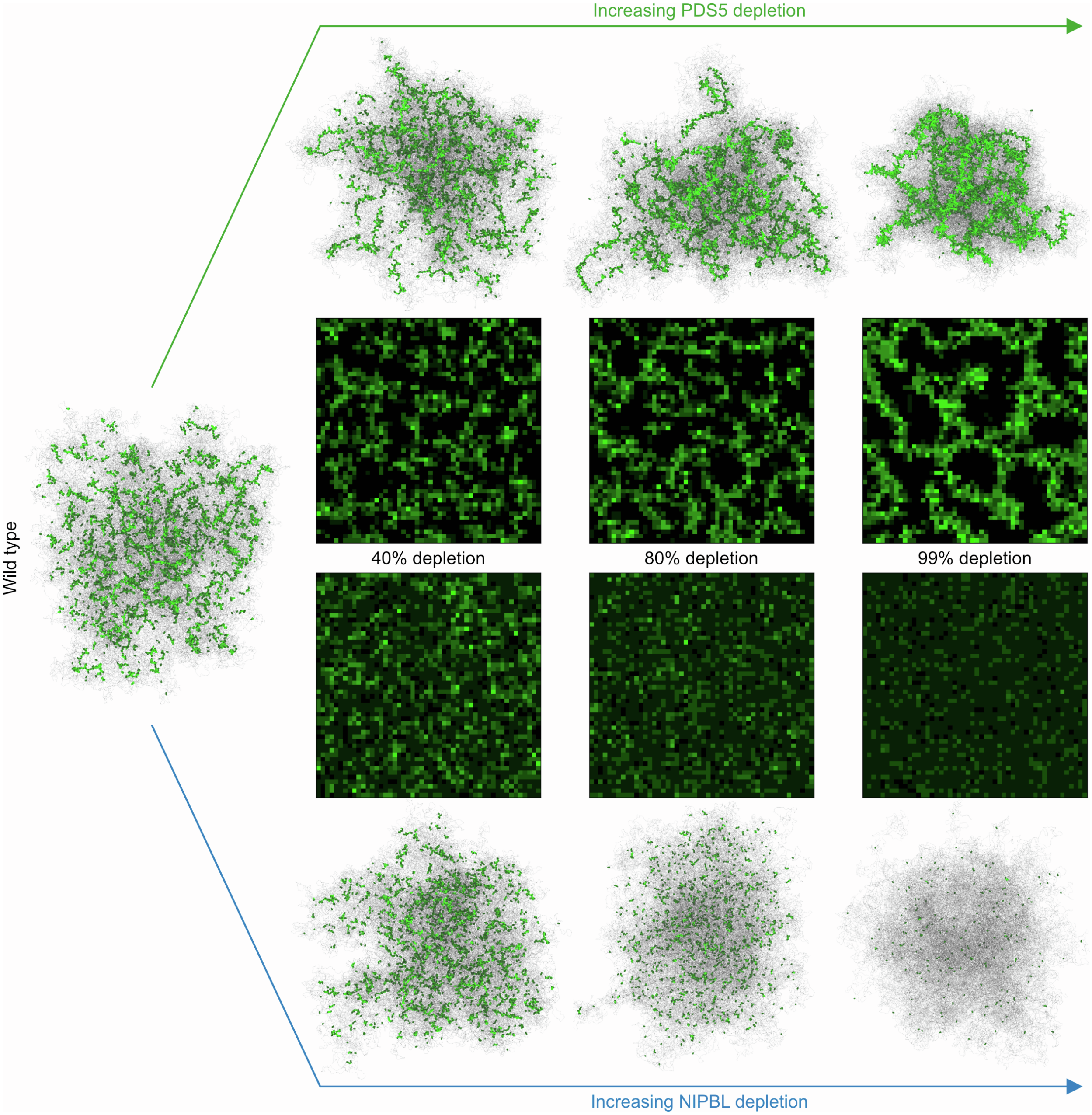

FIG. S10. **PDS5 and NIPBL depletion differentially affect chromosome structure (related to Fig. 5).**

Although PDS5 depletion leads to vermicelli phenotypes similar to ΔWAPL (c.f. Fig. 5b of the main text), the reduction in the loaded RAD21 population predicted in the case of NIPBL depletion leads to a gradual disappearance of the chromatin-associated cohesin signal.

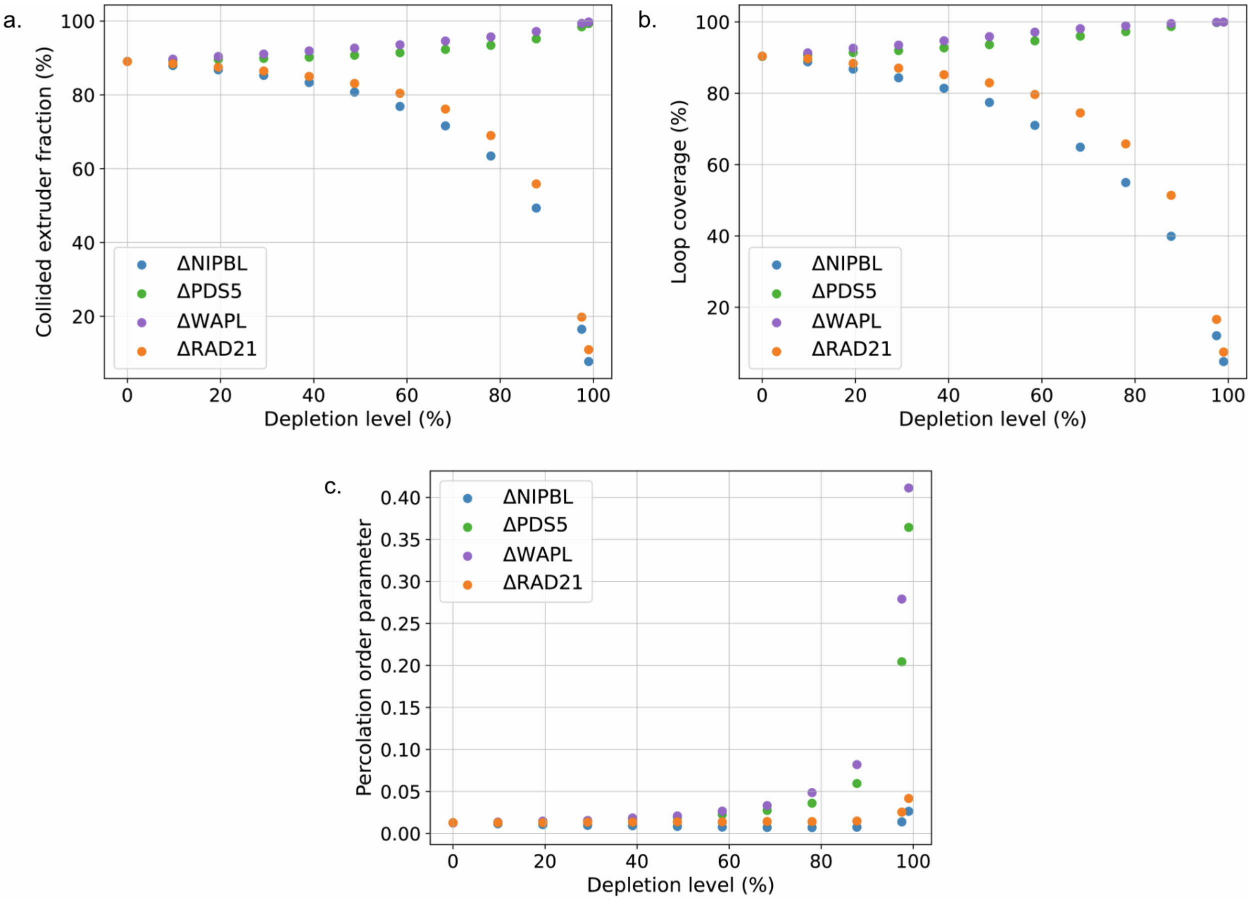

FIG. S11. **Alternative quantification of vermicelli formation (related to Fig. 5).**

a. Collided fraction of extruders as a function of depletion for the indicated factor. Collided fraction is calculated as the number of extruder legs directly adjacent to another extruder leg along the 1D lattice, divided by the total number of extruder legs, and are averaged over 5000 lattice conformations obtained across 5 independent simulations. The collided fraction increases with WAPL and PDS5 depletion, approaching 100%, and decreases for NIPBL and RAD21 depletion. **b.** Loop coverage, defined as the fraction of lattice sites that are encompassed by the two legs of any individual extruder. **c.** Cohesin percolation parameter, computed as the size of the largest cluster of collided extruders (as defined in (a)) normalized by the total number of loaded cohesins.
