# Supplementary material for: The physical chemistry of interphase loop extrusion
Source: Cell Genom. 2025 Dec 10;6(3):101098. doi: 10.1016/j.xgen.2025.101098 (PMC12985376; doi:10.1016/j.xgen.2025.101098)
Supplement: Methods S1. Technical details of the derivation of chemical-transition-rate mapping equations and proof of unicity of network equilibrium state, related to STAR Methods [file mmc2.pdf]

Cell Genomics, Volume *6*

Supplemental information

The physical chemistry of interphase loop extrusion

Maxime M.C. Tortora and Geoffrey Fudenberg

I. RATE MAPPING PROCEDURE

Using the notations of the main text, the chemical reaction network outlined in Fig. 1b explicitly reads as:

$$
\begin{aligned}
\mathrm{R}_{\mathrm{free}} + \mathrm{N}_{\mathrm{free}} &\xrightarrow{k_{\mathrm{on}}} \mathrm{RN}, \\
\mathrm{RN} &\underset{k_{\mathrm{RN}}}{\overset{k_{\mathrm{NR}}}{\rightleftharpoons}} \mathrm{R} + \mathrm{N}_{\mathrm{free}}, \\
\mathrm{R} + \mathrm{P}_{\mathrm{free}} &\underset{k_{\mathrm{PR}}}{\overset{k_{\mathrm{RP}}}{\rightleftharpoons}} \mathrm{RP}, \\
\mathrm{RP} + \mathrm{W}_{\mathrm{free}} &\underset{k_{\mathrm{WP}}}{\overset{k_{\mathrm{PW}}}{\rightleftharpoons}} \mathrm{RW} + \mathrm{P}_{\mathrm{free}}, \\
\mathrm{RW} &\xrightarrow{k_{\mathrm{off}}} \mathrm{R}_{\mathrm{free}} + \mathrm{W}_{\mathrm{free}},
\end{aligned}
$$

where the "free" subscript denotes species not bound to chromatin. In the framework of the law of mass action, the kinetic equations describing the time evolution of the free concentration of each cohesin subunit thus take the form of a system of coupled ODEs,

$$\frac{\partial [\mathrm{R}]_{\mathrm{free}}}{\partial t} = k_{\mathrm{off}} [\mathrm{RW}] - k_{\mathrm{on}} [\mathrm{R}]_{\mathrm{free}} [\mathrm{N}]_{\mathrm{free}}, \tag{1}$$

$$\frac{\partial [\mathrm{N}]_{\mathrm{free}}}{\partial t} = k_{\mathrm{NR}} [\mathrm{RN}] - k_{\mathrm{RN}} [\mathrm{R}] [\mathrm{N}]_{\mathrm{free}} - k_{\mathrm{on}} [\mathrm{R}]_{\mathrm{free}} [\mathrm{N}]_{\mathrm{free}}, \tag{2}$$

$$\frac{\partial [\mathrm{P}]_{\mathrm{free}}}{\partial t} = k_{\mathrm{PR}} [\mathrm{RP}] + k_{\mathrm{PW}} [\mathrm{RP}] [\mathrm{W}]_{\mathrm{free}} - k_{\mathrm{RP}} [\mathrm{R}] [\mathrm{P}]_{\mathrm{free}} - k_{\mathrm{WP}} [\mathrm{RW}] [\mathrm{P}]_{\mathrm{free}}, \tag{3}$$

$$\frac{\partial [\mathrm{W}]_{\mathrm{free}}}{\partial t} = k_{\mathrm{off}} [\mathrm{RW}] + k_{\mathrm{WP}} [\mathrm{RW}] [\mathrm{P}]_{\mathrm{free}} - k_{\mathrm{PW}} [\mathrm{RP}] [\mathrm{W}]_{\mathrm{free}}. \tag{4}$$

Let us consider the chromatin association/dissociation reaction of a generic species X,

$$\mathrm{X}_{\mathrm{free}} \underset{k_{\mathrm{off}}^{\mathrm{X}}}{\overset{k_{\mathrm{on}}^{\mathrm{X}}}{\rightleftharpoons}} \mathrm{X}_{\mathrm{bound}}.$$

In the limit of large excess of chromatin binding sites, the corresponding kinetic equation for the free X population reads as

$$\frac{\partial [\mathrm{X}]_{\mathrm{free}}}{\partial t} = k_{\mathrm{off}}^{\mathrm{X}} [\mathrm{X}]_{\mathrm{bound}} - k_{\mathrm{on}}^{\mathrm{X}} [\mathrm{X}]_{\mathrm{free}}. \tag{5}$$

Let us denote by $[\mathrm{X}]_{\mathrm{tot}} \equiv [\mathrm{X}]_{\mathrm{bound}} + [\mathrm{X}]_{\mathrm{free}}$ the total nuclear content of X. Assuming $[\mathrm{X}]_{\mathrm{tot}}$ to be constant throughout the G1 stage of the cell cycle, to which we restrict our current

study, the equilibrium bound fraction $f_{\rm X}$ may be obtained by solving Eq. (5) at steady state,

$$f_{\rm X} \equiv \frac{\left[{\rm X}\right]^{\rm eq}_{\rm bound}}{\left[{\rm X}\right]_{\rm tot}} = 1 - \frac{\left[{\rm X}\right]^{\rm eq}_{\rm free}}{\left[{\rm X}\right]_{\rm tot}} = \frac{k^{\rm X}_{\rm on}}{k^{\rm X}_{\rm on} + k^{\rm X}_{\rm off}}, \tag{6}$$

while the unbinding rate $k^{\rm X}_{\rm off}$ is related to the chromatin residence time $\tau_{\rm X}$ via

$$\tau_{\rm X} = \frac{1}{k^{\rm X}_{\rm off}}. \tag{7}$$

Substituting for species X the cohesin subunits RAD21, NIPBL, PDS5 & WAPL, a direct term-by-term comparison of Eqs. (1)–(4) with Eq. (5) yields

$$k^{\rm R}_{\rm on} = k_{\rm on}\left[{\rm N}\right]_{\rm free}, \tag{8}$$

$$k^{\rm R}_{\rm off} = k_{\rm off}\frac{\left[{\rm RW}\right]}{\left[{\rm R}\right]_{\rm bound}}, \tag{9}$$

$$k^{\rm N}_{\rm on} = k_{\rm RN}\left[{\rm R}\right] + k_{\rm on}\left[{\rm R}\right]_{\rm free}, \tag{10}$$

$$k^{\rm N}_{\rm off} = k_{\rm NR}, \tag{11}$$

$$k^{\rm P}_{\rm on} = k_{\rm RP}\left[{\rm R}\right] + k_{\rm WP}\left[{\rm RW}\right], \tag{12}$$

$$k^{\rm P}_{\rm off} = k_{\rm PR} + k_{\rm PW}\left[{\rm W}\right]_{\rm free}, \tag{13}$$

$$k^{\rm W}_{\rm on} = k_{\rm PW}\left[{\rm RP}\right], \tag{14}$$

$$k^{\rm W}_{\rm off} = k_{\rm off} + k_{\rm WP}\left[{\rm P}\right]_{\rm free}, \tag{15}$$

where we used $\left[{\rm N}\right]_{\rm bound} = \left[{\rm RN}\right]$, $\left[{\rm P}\right]_{\rm bound} = \left[{\rm RP}\right]$ and $\left[{\rm W}\right]_{\rm bound} = \left[{\rm RW}\right]$. Plugging in Eqs. (6) and (7), Eqs. (8)–(15) may be recast in the form, at chemical equilibrium,

$$\frac{1}{\tau_{\rm R}}\frac{f_{\rm R}}{1-f_{\rm R}} = k_{\rm on}\left(1-f_{\rm N}\right)\left[{\rm N}\right]_{\rm tot}, \tag{16}$$

$$\frac{1}{\tau_{\rm R}} = k_{\rm off}\frac{f_{\rm W}\left[{\rm W}\right]_{\rm tot}}{f_{\rm R}\left[{\rm R}\right]_{\rm tot}}, \tag{17}$$

$$\frac{1}{\tau_{\rm N}}\frac{f_{\rm N}}{1-f_{\rm N}} = k_{\rm RN}\left(f_{\rm R}\left[{\rm R}\right]_{\rm tot} - f_{\rm N}\left[{\rm N}\right]_{\rm tot} - f_{\rm P}\left[{\rm P}\right]_{\rm tot} - f_{\rm W}\left[{\rm W}\right]_{\rm tot}\right) + k_{\rm on}\left(1-f_{\rm R}\right)\left[{\rm R}\right]_{\rm tot}, \tag{18}$$

$$\frac{1}{\tau_{\rm N}} = k_{\rm NR}, \tag{19}$$

$$\frac{1}{\tau_{\rm P}}\frac{f_{\rm P}}{1-f_{\rm P}} = k_{\rm RP}\left(f_{\rm R}\left[{\rm R}\right]_{\rm tot} - f_{\rm N}\left[{\rm N}\right]_{\rm tot} - f_{\rm P}\left[{\rm P}\right]_{\rm tot} - f_{\rm W}\left[{\rm W}\right]_{\rm tot}\right) + k_{\rm WP}f_{\rm W}\left[{\rm W}\right]_{\rm tot}, \tag{20}$$

$$\frac{1}{\tau_{\rm P}} = k_{\rm PR} + k_{\rm PW}\left(1-f_{\rm W}\right)\left[{\rm W}\right]_{\rm tot}, \tag{21}$$

$$\frac{1}{\tau_{\rm W}}\frac{f_{\rm W}}{1-f_{\rm W}} = k_{\rm PW}f_{\rm P}\left[{\rm P}\right]_{\rm tot}, \tag{22}$$

$$\frac{1}{\tau_{\rm W}} = k_{\rm off} + k_{\rm WP}\left(1-f_{\rm P}\right)\left[{\rm P}\right]_{\rm tot}, \tag{23}$$

in which we substituted $[\mathrm{R}] = f_\mathrm{R}[\mathrm{R}]_\mathrm{tot} - f_\mathrm{N}[\mathrm{N}]_\mathrm{tot} - f_\mathrm{P}[\mathrm{P}]_\mathrm{tot} - f_\mathrm{W}[\mathrm{W}]_\mathrm{tot}$ based on the mass conservation of RAD21.

Using the experimentally-determined bound fractions f_X and residence times τ_X estimated from FRAP for each of the 4 relevant cohesin subunits, as well as the absolute G1 protein numbers $[\mathrm{X}]_\mathrm{tot}$ obtained as described in the main text, Eqs. (16)–(23) yield a linear system of 8 coupled equations involving the 8 unknown rates k_on, k_off, k_NR, k_RN, k_RP, k_PR, k_PW, k_WP governing the chemical reaction network (Fig. 1b). Eqs. (16)–(23) were inverted symbolically using the *SymPy* library, and the computed values for the rates k were plugged into Eqs. (1)–(4), which were then integrated numerically as described in the main text.

For the reaction network model described by Eqs. (1)–(4) ("bursty extrusion model"), the explicit expressions for the corresponding transition rates are as follows (c.f. Table S1),

$$k_\mathrm{on} = \frac{f_\mathrm{R}}{(1-f_\mathrm{N})(1-f_\mathrm{R})[\mathrm{N}]_\mathrm{tot}\tau_\mathrm{R}}, \tag{24}$$

$$k_\mathrm{off} = \frac{f_\mathrm{R}[\mathrm{R}]_\mathrm{tot}}{f_\mathrm{W}[\mathrm{W}]_\mathrm{tot}\tau_\mathrm{R}}, \tag{25}$$

$$k_\mathrm{NR} = \frac{1}{\tau_\mathrm{N}}, \tag{26}$$

$$k_\mathrm{RN} = \frac{f_\mathrm{N}[\mathrm{N}]_\mathrm{tot}\tau_\mathrm{R} - f_\mathrm{R}[\mathrm{R}]_\mathrm{tot}\tau_\mathrm{N}}{(1-f_\mathrm{N})[\mathrm{N}]_\mathrm{tot}\left(f_\mathrm{R}[\mathrm{R}]_\mathrm{tot} - f_\mathrm{N}[\mathrm{N}]_\mathrm{tot} - f_\mathrm{P}[\mathrm{P}]_\mathrm{tot} - f_\mathrm{W}[\mathrm{W}]_\mathrm{tot}\right)\tau_\mathrm{N}\tau_\mathrm{R}}, \tag{27}$$

$$k_\mathrm{RP} = \frac{f_\mathrm{P}[\mathrm{P}]_\mathrm{tot}\tau_\mathrm{R}\tau_\mathrm{W} + f_\mathrm{R}[\mathrm{R}]_\mathrm{tot}\tau_\mathrm{P}\tau_\mathrm{W} - f_\mathrm{W}[\mathrm{W}]_\mathrm{tot}\tau_\mathrm{P}\tau_\mathrm{R}}{(1-f_\mathrm{P})[\mathrm{P}]_\mathrm{tot}\left(f_\mathrm{R}[\mathrm{R}]_\mathrm{tot} - f_\mathrm{N}[\mathrm{N}]_\mathrm{tot} - f_\mathrm{P}[\mathrm{P}]_\mathrm{tot} - f_\mathrm{W}[\mathrm{W}]_\mathrm{tot}\right)\tau_\mathrm{P}\tau_\mathrm{R}\tau_\mathrm{W}}, \tag{28}$$

$$k_\mathrm{PR} = \frac{1}{\tau_\mathrm{P}} - \frac{f_\mathrm{W}[\mathrm{W}]_\mathrm{tot}}{f_\mathrm{P}[\mathrm{P}]_\mathrm{tot}\tau_\mathrm{W}}, \tag{29}$$

$$k_\mathrm{PW} = \frac{f_\mathrm{W}}{f_\mathrm{P}[\mathrm{P}]_\mathrm{tot}(1-f_\mathrm{W})\tau_\mathrm{W}}, \tag{30}$$

$$k_\mathrm{WP} = \frac{f_\mathrm{W}[\mathrm{W}]_\mathrm{tot}\tau_\mathrm{R} - f_\mathrm{R}[\mathrm{R}]_\mathrm{tot}\tau_\mathrm{W}}{f_\mathrm{W}[\mathrm{W}]_\mathrm{tot}(1-f_\mathrm{P})[\mathrm{P}]_\mathrm{tot}\tau_\mathrm{R}\tau_\mathrm{W}}. \tag{31}$$

For the combinatorial exploration of reaction networks, all possible permutations of bound cohesin state sequences were systematically generated using the *itertools* library, and the corresponding kinetic (Eqs. (1)–(4)) and rate-mapping (Eqs. (16)–(23)) equations were derived programmatically and similarly solved symbolically using *SymPy* to yield the associated network transition rates (Eqs. (24)–(31)).

II. EXISTENCE AND UNIQUENESS OF STEADY STATE

In the formalism of chemical reaction theory (Feinberg, 1995 [39]), the *deficiency* δ of a network is defined as:

$$\delta = n - l - s, \tag{32}$$

For the "bursty extrusion" network described in Sec. I, $n = 5$ and $l = 1$ are the respective numbers of nodes and connected components of the network. In Eq. (32), $s = \text{rank}(\mathcal{S}) = 4$ is the rank of the stoichiometric matrix $\mathcal{S}$ associated with Eqs. (1)–(4),

$$\mathcal{S} = \begin{array}{c} \begin{array}{cccccccc} [\text{R}]_{\text{free}} & [\text{N}]_{\text{free}} & [\text{P}]_{\text{free}} & [\text{W}]_{\text{free}} & [\text{R}] & [\text{RN}] & [\text{RP}] & [\text{RW}] \end{array} \\ \left[\begin{array}{cccccccc} -1 & -1 & 0 & 0 & 0 & 1 & 0 & 0 \\ 0 & 1 & 0 & 0 & 1 & -1 & 0 & 0 \\ 0 & -1 & 0 & 0 & -1 & 1 & 0 & 0 \\ 0 & 0 & -1 & 0 & -1 & 0 & 1 & 0 \\ 0 & 0 & 1 & 0 & 1 & 0 & -1 & 0 \\ 0 & 0 & 1 & -1 & 0 & 0 & -1 & 1 \\ 0 & 0 & -1 & 1 & 0 & 0 & 1 & -1 \\ 1 & 0 & 0 & 1 & 0 & 0 & 0 & -1 \end{array}\right] \end{array} \begin{array}{l} \\ \text{R}_{\text{free}} \to \text{RN} \\ \text{RN} \to \text{R} \\ \text{R} \to \text{RN} \\ \text{R} \to \text{RP} \\ \text{RP} \to \text{R} \\ \text{RP} \to \text{RW} \\ \text{RW} \to \text{RP} \\ \text{RW} \to \text{R}_{\text{free}} \end{array},$$

in which blue and red labels respectively denote forward and backward chemical transitions (c.f. Fig. 1b). Thus, $\delta = 0$, and the zero deficiency theorem guarantees that the network admits a unique equilibrium state, given a positive set of transition rates and total concentrations for each chemical species. Such rates may be uniquely determined from experimental data for each network structure and topology following the procedure in Sec. I (Eqs. (24)–(31)), implying that our model provides a one-to-one mapping between cohesin regulator levels and equilibrium state populations — and hence, their resulting steady-state cohesin extrusion kinetics (c.f. Methods).
